# Applications of Nanopipettes in Scanning Ion Conductance Microscopy for High-Spatial-Resolution Topographic Imaging and Sensing in Single Cells

**DOI:** 10.1021/acsmeasuresciau.5c00192

**Published:** 2026-02-10

**Authors:** Yusuf Muhammed, Ana B. Ramirez, Robert A. Lazenby

**Affiliations:** Department of Chemistry and Biochemistry, 7823 Florida State University, Tallahassee, Florida 32306, United States

**Keywords:** nanopipette, scanning ion conductance microscopy (SICM), scanning probe microscopy, single cell analysis, live cell imaging, nanoscale imaging, chemical sensing, biomolecule detection, biomedical research, bioanalytical

## Abstract

Membrane structures and cellular phenomena have been studied using scanning ion conductance microscopy (SICM). Conventional techniques for studying single cells, such as optical microscopy, fluorescence microscopy, electron microscopy (EM), and atomic force microscopy (AFM), have provided a wealth of information on the architecture of cell membranes, but they could potentially be invasive to live cells due to reasons including phototoxicity, electron beam damage, and cantilever-mediated damage to the cell membrane. While super-resolution approaches such as stimulated emission depletion (STED) microscopy have extended the capabilities of optical imaging, conventional optical microscopy remains limited by the diffraction limit in resolving intricate structures on cell membranes. In this review article, we discuss SICM as a technique that allows noninvasive imaging of live single cells in aqueous solutions, including cell culture media. We also discuss the fabrication and characterization of nanopipettes, advances in instrumentation and scanning regimes used in SICM, and applications of nanopipettes in the technique for topography mapping, high spatial resolution imaging, precise delivery of molecules to cells, biopsy, and surface charge measurements. We also discuss how nanopipettes are functionalized for applications in the simultaneous mapping of cell topography and high spatial resolution sensing, such as extracellular pH mapping. SICM has also been combined with scanning electrochemical microscopy (SECM) to enable the measurement of electroactive species at the cell membrane, and applied in cell surface charge mapping, where membrane charge is implicated in many cellular events. Advances in SICM imaging speed will allow the capture of fast cellular phenomena, and because the application of nanopipettes in SICM for high spatial resolution topographic imaging and sensing is still in its infancy, these developments could open new opportunities for imaging the distribution of analytes around live single cells.

## Graphical abstract

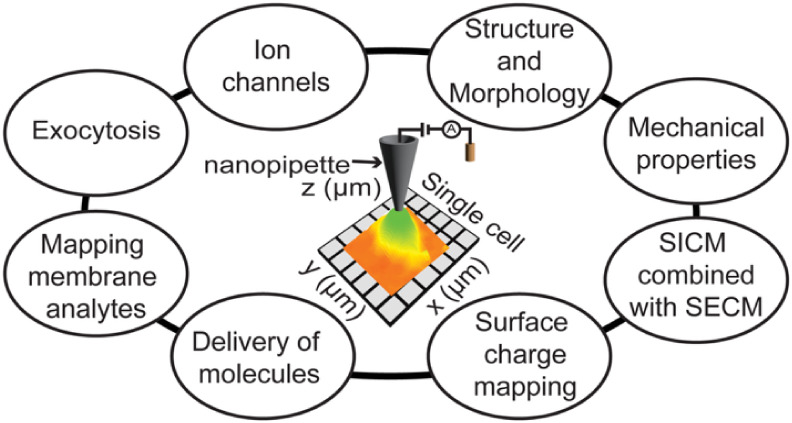

## 1 Introduction

Prior to the advancement of the field of nanopipettes, micropipettes were used to study cell electrophysiology, inject molecules into cells, and perform cell biopsies.^1–3^ The use of micropipettes led to advances in molecular and cellular studies, where analysis of cells in their native state with high resolution has been achieved.^4^ A key limitation of using micropipettes to perform measurements at or within cells is their potential to cause damage to cells, as a result of their relatively large size as compared to nanopipettes that are nanometer sized and considered noninvasive.^5^

The introduction of nanopipettes sparked greater interest in investigating the modification of nanopipettes for detecting different molecules such as DNA, RNA, amino acids, peptides, and proteins.^6^ Modified and unmodified nanopipettes have been widely employed as precision measurement tools in a range of cellular studies, to perform tasks such as injecting molecules into the cytoplasm, sampling cytoplasmic contents, measuring surface topography, and sensing biomolecules.^5^ Modified nanopipettes can be used for measuring analytes at single cells with high spatial resolution.^7,8^ The ultrasmall size of a nanopipette tip opening, together with its needle-shaped geometry, makes it suitable for precise analytical applications in scanning ion conductance microscopy (SICM).^9^ The development of nanopipettes has been motivated in part by the rapid expansion of cellular and molecular studies, where a versatile tool that can be precisely controlled or positioned close to a sample to give an accurate measurement is required.^10^

SICM is a scanning probe microscopy (SPM) technique that uses a nanopipette as the imaging probe. The technique was first described by Hansma and coworkers in 1989, and at that time it was primarily used for imaging flat polymer films.^11^ A major advance was made in 1997, when Korchev and coworkers demonstrated the use of the technique for imaging soft biological samples.^12^ The technique itself relies on using a nanometer sized borosilicate glass or quartz nanopipette probe, which allows nanometer resolution noninvasive imaging of single cell membranes in cell culture media, as demonstrated for a range of cell lines.^13^ Takahashi et al. revealed that nanopipettes are an important tool for use in SICM to spatially resolve membrane structures such as the formation and disappearance of endocytic pits.^14^ They further discussed that the success of high resolution timelapse imaging of cell membranes relies on fabricating nanopipettes with certain features such as thin wall thicknesses and small inner diameters. For cell imaging, nanopipettes are most frequently scanned in a hopping mode by repeatedly approaching the probe toward the sample surface until a current set point is reached, and then the probe retracts. This enables noninvasive mapping of complex structures on the steep slopes of the cell membrane. Refer to Section 2.3 for a more detailed discussion of hopping-SICM.^15^

Imaging techniques are important in deciphering biochemical processes such as cell differentiation, motility, changes in morphology, trafficking in and out of the cell membrane, and cell-to-cell communication. Besides topographical measurements, SICM can be combined with other techniques, such as electrochemical, fluorescence, and confocal microscopy, making it a crucial technique in the biomedical field.^16^ This review focuses on biological studies carried out using SICM, specifically on applications in single-cell imaging of membrane properties and the simultaneous mapping of cell membranes and measurement of analytes. We also describe nanopipette fabrication and characterization, SICM instrumentation, imaging resolution, and how SICM as a technique has evolved to image a range of biological samples, and the advances in instrumentation that have enabled such studies.

## 2 Experimental Setup and Imaging Modes of SICM

### 2.1 Fabrication and Characterization of Nanopipettes

Fabricating nanopipettes with the appropriate tip geometry is important for high resolution imaging of cell membrane structures.^14,17^ Nanopipettes are typically fabricated from quartz or borosilicate glass capillaries, using a heated-filament or laser-based pipet puller (Figure 1a). These instruments are capable of heating and pulling a single quartz or borosilicate glass capillary into two mirror-image twin nanopipettes, by heating a defined region to a temperature that exceeds the glass softening temperature. This pulling of the ends of the glass capillary along the longitudinal axis of the capillary tube generates two nanopipettes. The pulling process is carried out at a predefined velocity with a defined pulled length and a cooling period following the pull. The resultant nanopipette has a sharp taper at one end (Figure 1b), with an opening diameter and taper length that depend on the pulling parameters used.^18^

**Figure 1:**
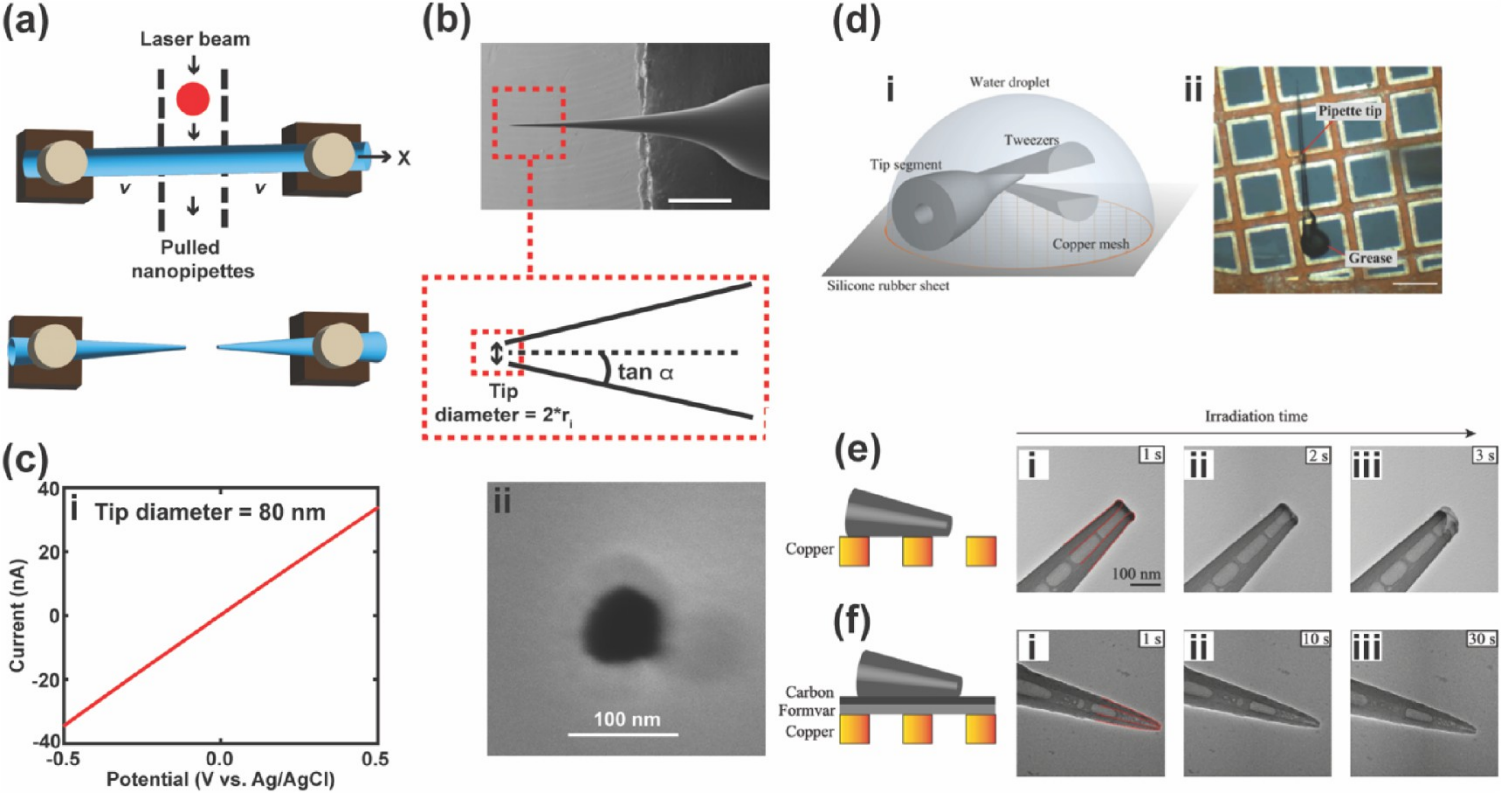
Fabrication and characterization of a nanopipette. (a) The principle of operation of a laser puller in generating two nanopipettes from a single quartz or borosilicate glass capillary tube. (b) SEM image of a nanopipette (scale bar = 500 μm) (top), and a schematic showing key elements of the probe geometry, where *r*_i_ is the inner radius, and α designates the inner half cone angle (bottom). (c) Characterization of an 80 nm diameter nanopipette tip using (i) cyclic voltammetry in 1.0 M KCl solution and (ii) SEM Figure 1c was adapted from ref^19^. Available under a CC-BY 3.0 license. Copyright 2024 Muhammed et al. Published by the Royal Society of Chemistry. (d) Tip segment of a nanopipette held in a droplet (i) and placed on a TEM grid (ii). (e) Nanopipette tip segment that was not supported by carbon Formvar, showing destruction of the tip (i–iii), and (f) nanopipette tip segment placed on carbon Formvar, showing no destruction of the tip (i–iii). Figures in parts d–f were adapted with permission from ref^20^. Copyright 2020 American Chemical Society.

Quartz glass capillaries are generally preferred for smaller diameter (e.g., 10 nm) pipet openings, due to its rigidity, lower dissipation factor (∼10^–4^) and low dielectric constant (3.8), while borosilicate glass capillaries are generally preferred for nanopipettes with diameters above 80 nm.^5^ During the fabrication of an SICM probe, adjustment of the laser puller pulling parameters such as heat (i.e., laser power), filament (the spread of laser scanning pattern), velocity (determines the movement of puller bar before the nanopipette is pulled), delay (defines the time in between hard pull and laser deactivation after pulling the nanopipette), and pull (determines pulling force), will play an important role in controlling the size and shape of a fabricated nanopipette.^5^ The heat generated by the laser puller to melt the capillary tube may be affected by ambient temperature and humidity. Cleaning of the glass capillary using piranha solution or ethanol to remove unwanted contamination before pulling is preferred for optimal performance.^21,22^

Optimization of the pulling parameters is required to achieve the desired size and geometry of the nanopipette, and there are many example programs described in the literature. For example, using the P-2000 Sutter laser puller, our group has fabricated probes for SICM with a diameter of ≤100 nm, using quartz glass capillaries with an outer diameter (OD) of 1.0 mm and inner diameter (ID) of 0.5 mm, and the following parameters: HEAT, 465; FILAMENT, 1; VELOCITY, 30; DELAY, 145; PULL, 175.^19^ We have also fabricated double barrel nanopipettes using a two-line program, and theta quartz capillary tubes (1.2 mm OD and 0.9 mm ID) to achieve a 100 nm tip diameter (each barrel), using the following parameters: line 1: HEAT, 560; FILAMENT, 2; VELOCITY, 30; DELAY, 145; PULL, 175, then line 2: HEAT, 570; FILAMENT, 2; VELOCITY, 20; DELAY, 125; PULL, 180. The pipet fabrication parameters listed are specific to the P-2000 laser puller used in our lab, and we note that parameters are influenced by the filament condition (when heated filaments are used), and environmental factors, including humidity and temperature. As such, these parameters should be used as starting points rather than fixed values.

After the fabrication of nanopipettes, characterization of the probe size and geometry is important (Figure 1c) to understand the achievable resolution of the probe and choose appropriate experimental parameters for imaging. The size of nanopipettes can be determined using electrochemical methods such as linear sweep voltammetry (LSV) or cyclic voltammetry (CV), performed in a two-electrode setup. One of the Ag/AgCl electrodes is held within a nanopipette filled with 1 M KCl, while the other electrode is a Ag/AgCl counter/reference immersed in 1 M KCl bulk solution outside the nanopipette. The CV generated (Figure 1ci) can be used to determine the tip inner radius, *r*_i_, calculated using eq 1,^23^ where α is the half cone angle, which is an important parameter that dictates the sensitivity of a nanopipette to the surface:

1 $$R_{p}=\frac{1}{\kappa \pi r_{i}\tan\alpha },\mathrm{where}{\;R}_{p}=\frac{V}{I}$$

The value *k* is the conductivity of the solution used, *V* is the voltage applied, and *I* is the current generated. The nanopipette resistance, *R*_p_, can be determined by sweeping the voltage in a voltammogram and measuring the ion conductance current, between an electrode placed inside the nanopipette and another placed in the bathing solution outside the nanopipette, in a solution of known conductivity. The gradient of the current–voltage curve will yield resistance (*R*_p_), in which the current should follow a linear relationship with potential (voltage) for high electrolyte concentrations, in which the size of the electrical double layer (EDL) is much smaller than the size of the nanopipette opening. A commonly used electrolyte concentration is 1.0 M. At lower salt concentrations (≲0.1 M), EDL overlap within the confined nanopipette tip region leads to current rectification and nonlinear voltammetric behavior.^24^

Scanning electron microscopy (SEM) has been widely used to examine and measure SICM pipet tips, providing detailed information on tip geometry, internal and external radii, and cone angle with nanometer-scale resolution, which is far superior to optical microscopy Figure 1cii. It enables accurate geometric characterization, which is essential for understanding pipet performance and in monitoring reproducibility. Sample preparation for SEM imaging is usually destructive, when the pipet tip is cut and/or coated with a conducting material (e.g., gold, carbon, or platinum), which can alter true dimensions and introduce uncertainty, especially for tips below ∼100 nm in diameter. The conductive coating thickness must be estimated and corrected for, but is often imprecisely known, and charging effects or sample damage may occur from the electron beam. These issues make SEM unsuitable for routine, nondestructive pipet evaluation.^25^

Transmission electron microscopy (TEM) is advantageous for characterizing nanopipettes, because it allows visualization of the probe’s internal geometry, including measurement of the wall thickness and the inner wall cone angle, with nanometer-scale precision. However, its use is often limited by beam-induced heating that can deform or damage the glass. Shigyou et al. presented improved, reliable methods for fabricating sub-10 nm quartz nanopipettes with high reproducibility and for characterizing their tip geometry using TEM under minimized electron-beam deformation.^20^ This approach included using optimized TEM imaging conditions with a low electron dose, and a practical sample preparation method using water droplets to position the tip on the TEM grid. Furthermore, formvar/carbon-coated TEM grids were used to enhance heat dissipation (Figure 1d–f), and a simple technique for mounting multiple nanopipette tip segments on a single TEM grid was described. TEM imaging of nanopipette tips was conducted at 200 kV using a minimum dose system (MDS) to reduce beam-induced damage. In this mode, the electron beam was activated only during 1-s image captures, allowing precise control of total electron dose and minimizing heat-induced tip deformation. These combined advances enabled direct and accurate measurement of the pore diameter, wall thickness, and cone angle, with TEM results consistent with electrical *I–V* characterization. Overall, their work established a robust, nondestructive TEM protocol for precise geometric analysis and fabrication control of sub-10 nm nanopipettes.^20^ Other studies have also reported the characterization (using TEM) and use of sub 10 nm diameter nanopipettes for SICM imaging.^26^ Despite TEM imaging having been optimized, it remains time-consuming and requires specialized equipment, limiting its scalability for routine nanopipette analysis.

Because small cell surface structures can be deformed when using thick walled glass nanopipettes for SICM imaging (Figure 2ai,ii, SICM image: 10 × 10 μm), Takahashi et al. investigated the protocol necessary for the successful fabrication of nanopipettes for high spatial resolution imaging with SICM.^14^ A nanopipette with a small aperture and a thin glass wall is necessary for improving the spatial resolution (Figure 2bi,ii, SICM image: 10 × 10 μm). Therefore, to fabricate a nanopipette for use in high resolution SICM, a borosilicate capillary with a thinner glass wall and a smaller inner diameter is desirable. To achieve this, it is necessary to preheat the borosilicate capillary tube before the final pulling.

**Figure 2:**
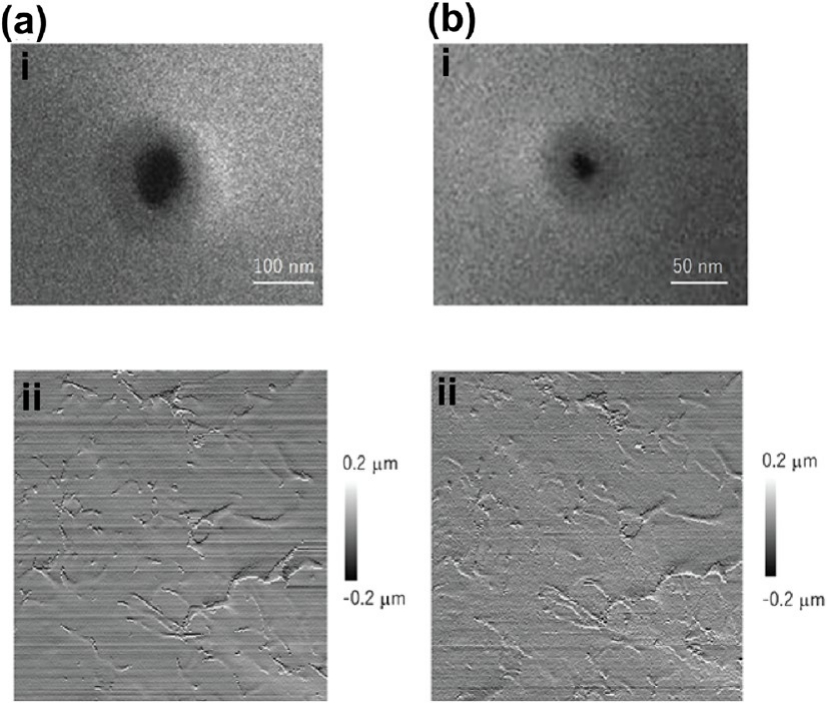
Fabrication of a nanopipette with a certain geometry dictates the resolution of an SICM image. (a) Using a conventional nanopipette geometry (i), the slope of the SICM image is captured with artifacts (ii). (b) Using a borosilicate nanopipette with smaller inner diameter and thin glass wall (i), the slope of the SICM image shows fewer artifacts (ii). SICM images in Figure b are 10 × 10 μm. Figure adapted with permission from ref^14^. Copyright 2023 American Chemical Society.

### 2.2 Instrumentation and Working Principle of SICM

An SICM setup can take multiple configurations, but will typically require: a potentiostat, used to apply a voltage and measure the current; piezoelectric positioners and controllers, that control the fine movement of the probe and/or sample in the *x*, *y*, and *z* directions; a coarse positioning system such as a micropositioner or a stepper motor; a glass nanopipette (scanning probe); and a personal computer and software to control the whole setup (Figure 3a).^27^

**Figure 3:**
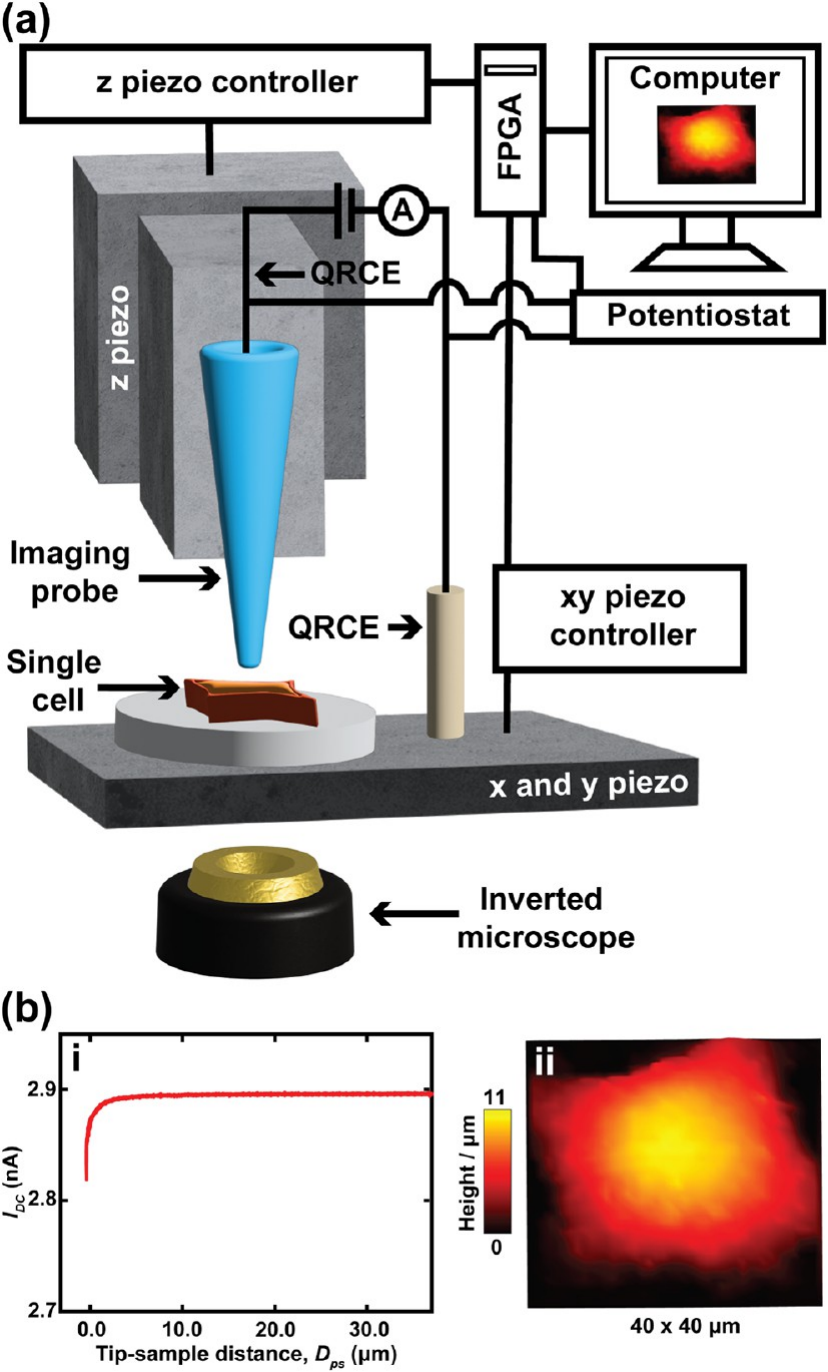
Basic components of SICM and how they are used in cell imaging. (a) Schematic showing the key instrumentation of a typical SICM setup. The basic setup in SICM comprises of a glass nanopipette probe positioned above the sample, an inverted microscope used to aid in tip positioning and sample viewing, a *z* piezoelectric positioner for translating the nanopipette in the vertical *z*-direction (controlled by *z* piezo controller), and *x* and *y* piezoelectric positioners (controlled by a piezo controller) for scanning the sample in the *x* and *y* directions, a potentiostat for applying a potential bias between the two QRCEs, and a computer with a field programmable gated array (FPGA) card, and a software interface for controlling the entire setup. The FPGA card is used to control the bias applied to the nanopipette, signals to the piezoelectric positioners, and in data acquisition and signal filtering.^28^ (b) (i) Approach curve used for setting feedback threshold prior to SICM imaging. (b) (ii) A typical example of an SICM image showing an A549 cell, obtained by hopping mode imaging. Figure 3b was adapted from ref^19^. Available under a CC-BY 3.0 license. Copyright 2024 Muhammed et al. Published by the Royal Society of Chemistry.

An inverted optical microscope equipped with a camera is a necessary addition for studies of transparent samples such as live cells, to aid in probe and sample positioning and visualization.^15,29,30^ The SICM setup is interfaced with personal computer software to control tip positioning and current measurements. Several software platforms are available, including Ionscope image software^31^ and LabVIEW. An open-source program control software written in LabVIEW, Warwick Electrochemical SPM (WEC-SPM), has been reported by McKelvey et al.^28^

To use SICM for topography measurements, a glass nanopipette probe is filled with an electrolyte solution such as KCl, then a silver chloride coated silver wire (Ag/AgCl) serves as a quasi-reference counter electrode (QRCE), which is back inserted into the nanopipette. Ag/AgCl electrodes are chosen because of their stable potential, and they do not contaminate the cell culture media during imaging. In nanopipettes, the current generated is typically in the nanoampere or subnanoampere range, so a QRCE can easily sustain the required current without polarization due to minimal ohmic drop, making a separate counter electrode unnecessary. After the nanopipette electrode is prepared, it is then mounted to a *z*-piezoelectric positioner. The tip of the nanopipette is submerged into the electrolyte solution bathing the sample, followed by the insertion of a second Ag/AgCl QRCE into the same bathing solution.^32,33^ Simultaneous movement of the probe downward in *z*-direction while applying a bias potential between the two electrodes results in a change of the steady-state ionic current, *I*(*D*_ps_), when the tip is close to a substrate surface (eq 2):^34^

2 $$I\left(D_{\mathrm{ps}}\right)=\frac{V}{R_{p}+R_{z}}\approx I_{\infty }{\left(\frac{1+\frac{3}{2}\ln\left(\frac{r_{o}}{r_{i}}\right)r_{i}r_{e}}{hD_{\mathrm{ps}}}\right)}^{-1};I_{\infty }=\frac{V}{R_{p}}$$

The magnitude of the ionic current, *I*_*∞*_, is determined by *R*_*p*_ (eq 2), *R*_*z*_ (the access resistance, affected by the distance from tip opening to substrate surface), *R*_s_ (the resistance of the solution), *D*_ps_, and *h*, which are defined as the tip–substrate distance and the height of the pipet relative to its base, respectively. When the nanopipette probe moves toward the surface and the current is measured, the resultant plot is called an approach curve (Figure 3bi). As reported by Zhu et al.,^35^ according to a proposed model by Nitz et al.,^34^ if the tip-to-substrate separation distance decreases, the access resistance will increase based on the tip geometry.^36^ Specifically, if the separation distance is less than the diameter of the tip, the flow of ionic current will decrease due to an increase in the access resistance (eq 3).^36^

3 $$R_{z}\approx \frac{\frac{3}{2}\left(\frac{r_{o}}{r_{i}}\right)}{\kappa \pi D_{\mathrm{ps}}}$$

This change can be used to detect and map the sample topography (Figure 3bii) based on a defined feedback set point, which is usually a 0.6 to 2% reduction of the bulk steady state current (*I*_∞_).^37^ The relationship between the ionic current and the distance of the glass nanopipette to the substrate in the *z*-direction is described by equations proposed by Nitz et al.^34^ The *R*_*z*_ depends on the ratio of the outer radius (*r*_0_) to *r*_i_, the tip–sample distance (*D*_ps_), and the *k* of the bulk electrolyte solution. The change in current (*I*) due to the change in *D*_ps_, can be calculated from *R*_*p*_, *R*_*z*_, and the bias voltage (*V*) applied between the two QRCEs.^34,35,38^

A more detailed approach curve equation was developed by Rheinlaender and Schäffer, which was derived from a finite element method (FEM) model analysis.^38^ Their studies still proposed that the resistance of current between the QRCE inside the nanopipette and the QRCE in the bulk solution is comprised of *R*_*p*_ and *R*_*z*_. Combining *R*_*p*_ (from eq 2), *R*_*z*_ (from either eqs 4 or 5), and Ohm’s law will result in a more accurate expression for the estimation of nanopipette distance as a function of change in the current *I*(*D*_ps_) (eqs 6 and 7), where *I*_∞_ is the bulk current.^38^ Despite the fact that the Nitz et al. model is widely used, the Rheinlaender model is preferred as the modeled approach curve fits better with experimental curves.

4 $$R_{z}\left(\frac{D_{\mathrm{ps}}}{r_{o}}\leq 0.2\right)\approx \frac{\ln\left(\frac{r_{o}}{r_{i}}\right)}{\kappa \pi D_{\mathrm{ps}}}$$

5 $$R_{z}\left(\frac{D_{\mathrm{ps}}}{r_{o}}\geq 0.2\right)\approx \frac{1}{4\kappa r_{i}}{\left(1+0.2\left(\frac{r_{o}}{D_{\mathrm{ps}}}\right)r_{i}r_{e}\right)}^{1.2}$$

6 $$I\left(D_{ps}\right)=\frac{V}{R_{p}+R_{z}}\approx I_{\infty }\frac{R_{p}+R_{z}\left(D_{ps}\to \infty \right)}{R_{p}+R_{z}\left(D_{ps}\right)}$$

7 $$I_{\infty }=\frac{V}{R_{p}+R_{z}\left(D_{\mathrm{ps}}\to \infty \right)}\approx \frac{\kappa Vr_{i}}{\frac{1}{\pi \tan\alpha }+\frac{1}{4}}$$

The values *R*_*p*_ and *R*_*z*_ are important for the resolution of the SICM image. The ion conductance current, which depends on tip–sample distance, is important in obtaining the spatial variation of an interface, and avoiding nanopipette tip damage during imaging.^35^ Additional discussion on the theory of nanopipettes has been provided by Li and Li.^39^

Since the inception of SICM, there have been several attempts to improve the capabilities of the technique. The conventional mode uses the principle that the application of a bias voltage between the external QRCE and the QRCE inside the nanopipette, results in the generation of an ion conductance current, *I,* which is inversely proportional to the total resistance (*R*_T_), and directly proportional to the bias voltage (*V*_B_) (eq 8).^40^ A study by Kim et al. described the development of an alternative setup, to overcome the low signal-to-noise ratio (SNR) and bandwidth trade-off in conventional SICM.^40^ In the alternative setup, the ion current, *I*, is converted to an output voltage (*V*_0_) by a transimpedance amplifier, which is directly proportional to the feedback resistance (*R*_F_) (eq 9). The transimpedance amplifier is associated with thermal noise, *T*_N_, (eq 10), which depends on the Boltzmann constant, *k*_B_ = 1.38 × 10^–23^ J K^–1^, temperature, *T*, and bandwidth, *BW*. Therefore, the improvement of the SNR depends on setting the value of *R*_F_ high, since the gain current increases as the *R*_F_ increases, and (*R*_F_)^1/2^ is proportional to thermal noise (*T*_N_). This results in low bandwidth and high SNR, which was shown to improve the resolution of SICM, demonstrated in the imaging of L929 fibroblast cells.

8 $$I=\frac{V_{B}}{R_{T}}$$

9 $$V_{O}=R_{F}.I$$

10 $$T_{N}=\sqrt{4.R_{F}.BW}$$

Biphasic SICM (BP-SICM), developed by Choi and Baker, uses a double barrel nanopipette filled with 1 M KCl, and SICM is carried out in a low conducting medium (such as a mix of KCl, deionized water, and ethanol).^41^ Both barrels of the nanopipette have a Ag/AgCl electrode inserted into them, and one of the barrels serves as the working electrode, while the other wire in the second barrel serves as the reference/counter electrode. Application of a potential bias to the working electrode will result in the flow of current between the two-barrel electrodes. This generates a zone of higher ion concentration between the tip and the interface of the bath solution. Since the ion current is useful in generating current feedback, it is thus suitable for imaging. This technique gives an approach curve with different features from the conventional SICM, in which as the probe is translated toward the surface, the current first increases, then reaches a steady state current before it starts decreasing. The rise in current is due to the decrease in ion diffusion into the bath solution that occurs as the *z* distance decreases. This current increase has been used to clearly image collagen fibrils using positive feedback SICM with 7%, 14%, and 21% current increase set points. Using a typical feedback magnitude of conventional SICM (i.e., a 1 to 2% increase) for imaging in positive feedback in BP-SICM resulted in poor resolution of collagen fibril topography. The decrease in current of the BP-SICM approach curve can also be used for traditional negative feedback imaging using 0.9% less than the bulk current, with a better resolution than using positive feedback. A recent study reported that using a double barrel probe, where one barrel is blocked using 100 nm diameter polystyrene beads (which generates a capacitive current) and the other barrel is open (with both capacitive and resistive current), allowed an improvement in ion current by reducing capacitive current through subtraction of current of the open barrel from the occluded one, allowing imaging of complicated surfaces rich in negative charge, like chromosomes.^42^

Ida et al. used low capacitance (6.8 μF for *x*,*y* and 1.4 μF for *z*) and high-resonance-frequency piezo stages (2.5 kHz for *x*,*y* and 6.2 kHz for *z*) to perform high speed imaging, where the SICM was modified with an algorithm to perform backsteps when the cell height is encountered (Figure 4ai).^43^ This method was successfully used to map the dynamics of microvilli on A431 cells and revealed the disappearance of microvilli after stimulation with epithelial growth factor (EGF) to buffer the cell tension during migration (Figure 4aii–vi).

**Figure 4:**
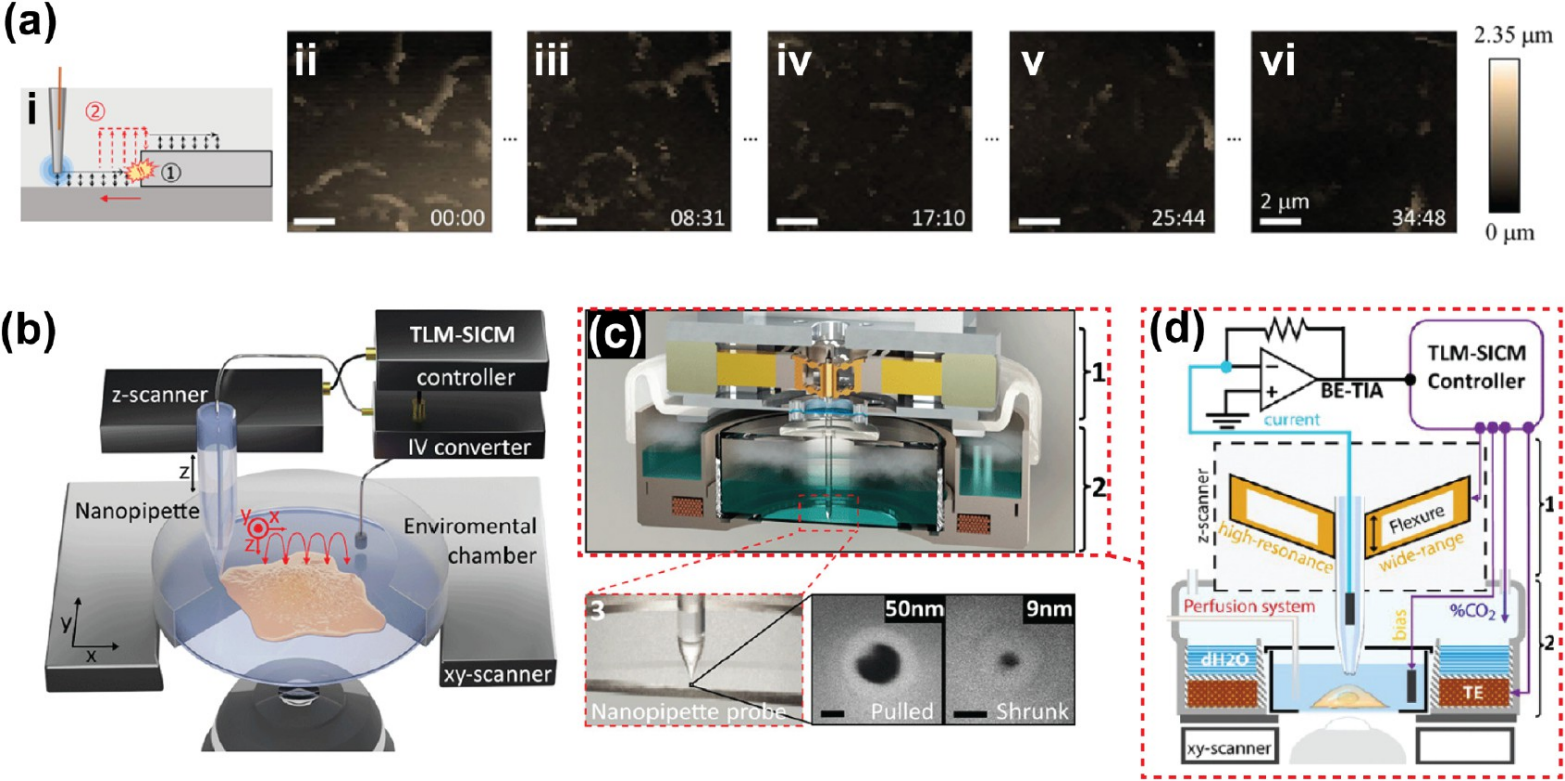
Advancement in SICM instrumentation for time-lapse studies. (a) Time-lapse SICM setup (i) and dynamic topography images (ii–vi). Figure was adapted with permission from ref^43^. Copyright 2017 American Chemical Society. (b) The setup for time-lapse microscopy (TLM)-SICM imaging of small region and whole cell topography, where (c) shows actuator with high bandwidth and large range, in region 1, and the CO_2_ chamber, in region 2. (d) A schematic of the labels of various SICM components shown in part c. Figures in parts b–d were reproduced with permission from ref^44^. Available under a CC-BY-NC-ND 4.0 license. Copyright 2021 Leitao et al. Published by the American Chemical Society. https://pubs.acs.org/doi/10.1021/acsnano.1c05202.

Recently, there has been further advancement in the performance of SICM to allow time-lapse measurement of a variety of cellular events beyond microvilli, as reported by Leitao et al. (Figure 4b).^44^ A key challenge during the hopping mode of SICM imaging (vide infra) is that after the nanopipette approaches a substrate surface and the current decreases to the set point, the *z*-piezo controller retracts the nanopipette at a very fast rate. When this movement speed is set as fast as possible, this results in an inertia that excites the *z*-piezo actuator resonance frequency, and this excitation is even higher when the approach velocity of the pipet is also high. To address this problem, *z*-piezo actuators with a high resonance frequency are used, at the expense of the *z*-piezo range. Imaging mammalian cells typically requires a long piezo actuator range, so there is a trade-off between resonance frequency and the actuator range. These challenges were addressed by Leitao et al. using high bandwidth developed from bandwidth-extended transimpedance amplifiers (BE-TIAs), and a large range *z* piezo actuator (22 μm), and the trade-off was resolved by adding adaptive gain to the piezo motion as a function of the current interaction curve, which slows the nanopipette before reaching the set point.^44^ Also, in the same work the authors introduced a custom-built CO_2_ incubator into the SICM setup to maintain cell viability during long-term imaging. This advancement in SICM enabled the measurement of time-lapse events such as endocytosis, micropinocytosis, mitosis, bacterial infection, and cell differentiation in cancer cells over an extended period. This development in time-lapse-SICM could potentially offer insight into how cells interact for infection, immunology, and cancer research, where time-lapse measurements with high temporal resolution are required.^45–48^

More advances have been made in the field of SICM on coupling the technique with spectroscopy, such as tip-enhanced Raman spectroscopy (TERS).^49,50^ This advancement could contribute to the detection of Raman scattering of analytes on the surface of materials and single cells. Interestingly, optogenetic SICM (opto-SICM) was developed to study cell–cell contact, which is remarkable progress in cell studies.^51^ The combination of super-resolution optical fluctuation imaging with SICM (SICM-SOFI) have allowed the correlative studies of membrane structure (actin) dynamics.^52^ SOFI offers high resolution imaging of cells without the concern of phototoxicity and diffraction limit, while SICM enables noninvasive imaging. This advancement has provided high resolution image of cytoskeleton in the cell membrane. Overall, the major limitation of SICM is low throughput, where one cell is mapped at a time. However, a recent study has introduced macro-SICM using long scan range piezo where multiple cells were mapped at the same time.^53^ Despite this improvement, the resolution of the technique is compromised.

### 2.3 Scanning and Feedback Modes of SICM

SICM can be operated in various scanning modes, that describe the motion of the tip, such as the constant distance mode (Figure 5a) and hopping mode (Figure 5b), or in various feedback modes (i.e., current used to sense the substrate surface), including bias modulated (BM) mode (Figure 5c), distance modulated/alternating current (AC) mode (Figure 5d), and direct current (DC) mode (Figure 5a,b). The type of current generated, the voltage applied, and the nature of the movement of the nanopipette probe in the *z*-direction will determine the mode of SICM in operation.

**Figure 5:**
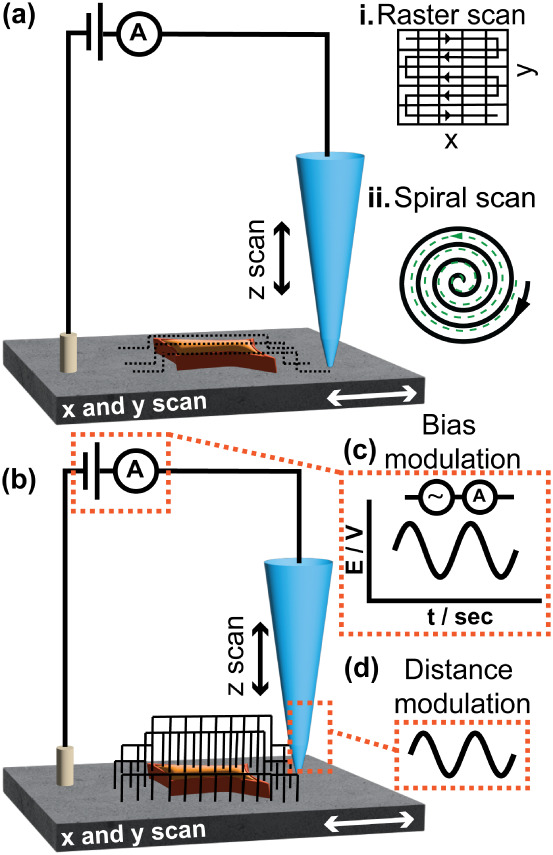
Schematic showing the modes of operation of SICM. (a) Constant distance, where the scanning probe continuously adjusts the *z* position to maintain a constant distance between the probe and the sample, while the sample is scanned laterally in the *x* and *y* directions. The probe scans in a raster (i) or spiral (ii) fashion. (b) Hopping mode, where the probe approaches a defined position above the sample, detects the surface, then retracts, and then moves laterally to a new position (i.e., pixel). (c) Bias modulated mode oscillates the tip voltage over time, and (d) AC mode applies a frequency to the micropositioner resulting in the oscillation of the tip position. This generates an AC that is detected by a lock-in amplifier and generates a signal to control the tip–sample distance.

During the development of SICM, Hansma et al. used the technique in constant distance mode to image machined plastic pieces using an eyedropper as the probe.^11^ The constant distance mode of SICM (Figure 5b) involves the movement of the glass nanopipette continuously to maintain a constant value of current, while the sample is scanned in the *x* and *y* directions. This lateral motion can be performed either in a raster (line-by-line parallel scan in a rectangular pattern) or spiral (circular) motion. The spiral pattern is preferred when fast scanning is to be achieved because it provides the advantage of higher angular velocity due to its continuous and smooth trajectory.^54^ Constant distance mode allows for faster imaging but is less suitable for samples with large gradient surfaces. Hopping-mode SICM, developed by Novak et al.^55^ was used to overcome the challenges of crashing the nanopipette tip in the constant distance mode during the imaging of convoluted surfaces (Figure 5b), for example, cell membranes. In this mode, the nanopipette tip is approached toward a surface (in the *z*-direction) at a particular position until a feedback current set point is reached, then the tip retracts by a preassigned distance and is laterally moved to the next position (i.e., next pixel of the image). This process is repeated until the topography of the scan area is obtained. While this mode of SICM is typically slower than constant distance imaging, it allows for scanning steep interfaces and topographically challenging substrates. Other modes were also developed to image convoluted surfaces, such as backstep-pulse current mode, developed by Happel et al.,^56^ and standing mode, developed by Takahashi et al.,^57^ however, the hopping mode has received the most attention in SICM for imaging live single cells.

Most of the recent advances in using SICM for biological studies use the hopping mode.^42,46^ The slow temporal resolution may affect the ability to capture ultrafast events occurring on the cell membrane. This is despite the improvement of the hopping speed to a pixel rate of 11 kHz by Simeonov and Schäffer, where dynamic topographic images were captured in seconds.^59^ Still, there are many cellular events occurring in milliseconds, such as changes in action potential in neurons, cytoskeleton activity near the cell membrane,^60^ and cytochrome c mediated electron transfer.^37,51,61–67^ Further improvement in the speed of hopping mode for ultrafast imaging could open new opportunities for biomedical applications of the technique to study fast cellular kinetics at faster time scale.

Among the feedback modes, the DC mode is the simplest, in which a bias voltage is applied between the QRCE in the nanopipette and the QRCE in the bulk electrolyte solution. This results in the generation of a DC, which is used for the feedback to detect the sample surface and maintain a constant distance between the glass nanopipette probe and the surface. A key drawback is that the current may drift in the duration of the scan.^40^ To overcome this, the bias applied between the QRCE of the nanopipette and the QRCE of the bulk electrolyte can be oscillated, termed as bias modulated (BM)-SICM, resulting in the generation of an AC signal (Figure 5c).^68^ Alternatively, the AC mode (distance modulated) involves the oscillation of the probe position through the *z* piezoelectric positioner, which results in an oscillating current (i.e., AC) when the tip–sample separation distance is small (Figure 5d). A lock-in amplifier is used to detect the changes of AC amplitude and phase due to the local ionic environment.^69^ The AC-SICM mode typically uses the application of a frequency of ∼100–1000 Hz to oscillate the *z* piezoelectric positioner holding the nanopipette, which generates a signal that is more sensitive to the presence of the surface of the sample, without sensitivity to surface charge.^70^ The basic principle of a lock-in amplifier involves the measurement of the amplitude, *V*_0_, of the sinusoidal voltage, *V*_in_(*t*), (eq 11), and phase of an input signal (for example a signal coming from the voltage applied to a nanopipette) with respect to a reference signal, typically generated by the lock-in. This is important in a situation where the signal of interest is masked by noise, *V*_n_(*t*), or signals of other frequencies (eq 11). The lock-in amplifier uses the frequency of the reference signal to locate the signal of interest from the noise, and measure its amplitude.^71^ Hence, in AC mode, the noise is eliminated to extract the signal (eq 11).^72,73^ The drawback of AC mode is the slow operation speed, which is due to the limitation of the piezoelectric actuator resonance frequency and the need for extraction of the AC signal using a lock-in amplifier.^17^

11 $$V_{\mathrm{in}}\left(t\right)=V_{o}\cos\left(w_{0}t\right)+V_{n}\left(t\right)$$

### 2.4 Resolution and Image Acquisition in SICM

The spatial resolution of SICM scanning during topography measurement depends on two factors: the size of the tip, and the imaging parameters such as the hopping distance or distance between lines of a constant distance mode scan (Figure 6a).^74,75^ For lateral resolution (i.e., the distance at which two different objects are resolved clearly from each other in an image), the probe size will determine the resolution, and it was claimed by Rheinlaender and Schäffer to be approximately three times the tip inner radius (3*r*_i_), using finite element method (FEM) modeling.^74^ However, there has been a lack of consensus regarding the lateral resolution of SICM. For example, Weiber and Baker reported the lateral resolution to be 0.5*r*_i_,^76^ while Del Linz et al. estimated it to be 2*r*_i_.^77^ However, to achieve this resolution, the user should choose imaging parameters appropriate for the tip size being used. Besides spatial resolution, the temporal resolution will be dictated by imaging parameters such as the approach rate, retract rate, lateral scan rate, hopping distance, and the dimensions of the area to be scanned. To increase the spatial resolution, a smaller hopping distance should be used, as this results in more approaches (i.e., pixels) per unit area (Figure 6b). Faster approach rates, lateral scan rates, and retract rates of SICM increase the speed of the SICM scanning, without affecting resolution. In the constant distance mode, there will generally be a greater number of pixels in the dimension along a line (e.g., *x* direction) than the dimension between lines (e.g., *y* direction), when using a raster scan pattern. Axial resolution of SICM has been reported as low as 5–10 nm.^44,52^

**Figure 6:**
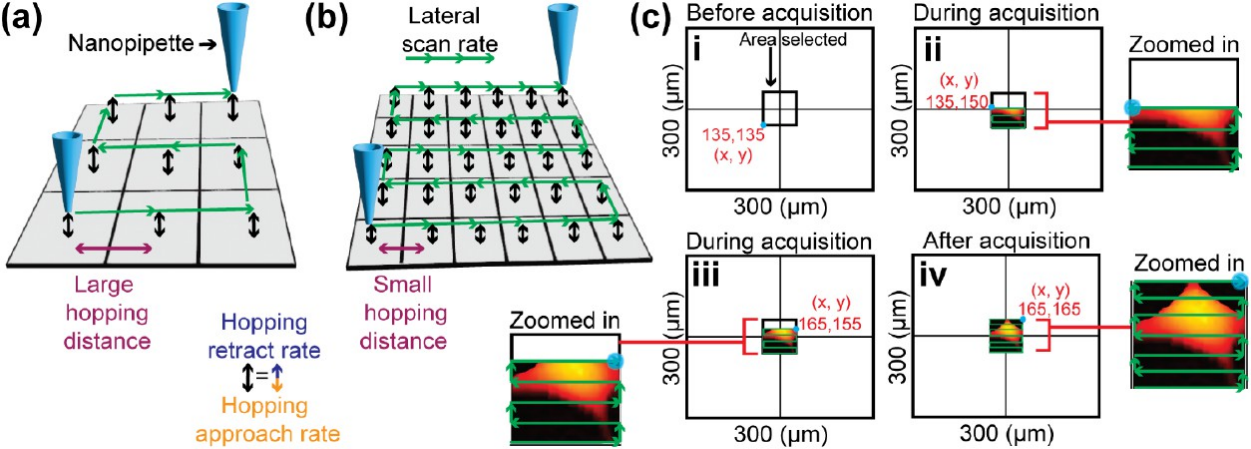
A schematic showing the key scanning parameters used for SICM imaging that dictate spatial and temporal resolution. The lateral resolution in hopping SICM is depicted for (a) low and (b) high spatial resolution imaging. (c) The acquisition of an SICM image. In this example, the *x* and *y* piezo have a scan range of 300 μm × 300 μm. (i) Initial state before acquisition. (ii, iii) Intermediate stages during acquisition. (iv) Final image corresponding to a 30 μm × 30 μm scanned area, as shown in the inset labeled “after acquisition.”.

To image cells with SICM, typically a Petri dish containing 2D surface adhered cells is placed on the *xy* piezo stage, while the nanopipette is mounted on the *z* piezo. Note that in this setup, the *z*-piezo is decoupled from the *x* and *y*, although other configurations are possible. The chosen scan area depends on the size of the cell or the specific region of interest. For instance, to record the topography of an entire cell over a 30 μm × 30 μm area using an *x* and *y* piezo with a 300 μm range in each direction (Figure 6c), the starting coordinates are chosen. With the *x* and *y* piezo center position coordinates of 150 μm, 150 μm, the nanopipette can begin the scan at 135 μm, 135 μm *x*,*y* coordinates (Figure 6ci). It then moves along the *x*-direction to scan the first line, in a pixel-by-pixel manner. After completing a line, the probe shifts to the next *y*-position and repeats this process. The scanning continues in this manner until the final position is reached (i.e., 165 μm, 165 μm coordinates in this example) (Figure 6civ).

A deep-learning assisted SICM approach has been developed to improve the temporal resolution of SICM, where nanoscale imaging is accelerated by skipping selected scan lines and reconstructing the missing data using a partial convolutional neural network (partial-CNN).^78^ The model restores skipped regions with high precision, which outperforms linear interpolation and standard CNNs, and makes imaging acquisition 30–63% faster without loss of structural details. This method enables real-time SICM imaging of dynamic biological processes and can also correct scan artifacts such as line scars. Recently, another study introduced super-resolution (SR)-SICM, that enhances the resolution and accuracy of conventional SICM.^79^ SR-SICM was developed by incorporating a computational deconvolution process based on simulated pipet point-spread functions (PSFs), which model the ion current distribution around the nanopipette tip. This approach effectively removes blurring artifacts caused by the finite pipet size, thereby surpassing the lateral resolution limits of standard SICM. Demonstrations on nanostructures, including lipid bilayers, amyloid peptide fibrils, and DNA nanostructures show that SR-SICM achieves near single-molecule resolution under physiological conditions, without damaging samples. Furthermore, the authors report that SR-SICM can be used with any SICM data set using a downloadable user-friendly software, making the technique accessible for broad applications in single molecule studies using SICM.

## 3 Mapping Structures and Biomolecules on the Surfaces of Cell Membranes

### 3.1 Revealing Cell Membrane Proteins, Ion Channels, Endocytic Pits, and the Cytoskeleton

AFM has excellent nanometer lateral and axial resolution; however, the imaging of eukaryotic cells can suffer from contact between the sample and the cantilever tip, potentially damaging membrane structures. Live cell imaging can be carried out using fluorescence microscopy, but nanoscale topological details at the surface of live cells cannot be examined, making it challenging to be used for imaging membrane structures. SICM is suitable for imaging of membrane structures, relative to the membrane topography, in live cells.^35,80,81^ For example, the topography of membrane protein complexes on the surface of spermatozoon cells has been reported using SICM.^13^ The spermatozoon membrane is organized into distinct topographical domains, such as the anterior acrosome region, equatorial segment (and subsegment), postacrosome, midpiece, and principal piece. High resolution SICM of the equatorial segment and subsegment of the acrosome-reacted cells revealed the height and width of projecting membrane protein molecules on the membrane, proposed to be reminiscent of the intramembrane particles that play crucial roles as ion channels and receptors. Some of these molecules are stable after taking images of the equatorial segment and subsegment, at 10 min intervals.

Another interesting structure of the cell membrane mapped with SICM is clathrin-coated pit (Figure 7a), which mediates endocytosis studies.^82^ Endocytosis is a cellular process where the cell membrane invaginates to form a vesicle that internalizes extracellular material into the intracellular compartment, mediated by proteins such as clathrin and dynamin.^83^ Techniques such as scanning transmittance microscopy, electron microscopy, confocal microscopy, and total internal reflection (TIR) microscopy have been applied in studies of endocytosis. TIR microscopy cannot be used in the study of apical membranes, and information about single endocytic pits cannot be obtained using confocal microscopy. The precise location of the endocytic pit, relative to the cell membrane, cannot be visualized with fluorescence microscopy. Using SICM combined with surface confocal microscopy, endocytosis has been studied by detecting endocytic invagination on the surface COS-7 cells engineered with clathrin labeled with green fluorescent protein (GFP).^84^ The clathrin protein mediates the formation of a membrane invagination, resulting in the formation of a pit on the cell surface. In the SICM experimental setup, while mapping the topography of the endocytic pit relative to the cell membrane, clathrin-GFP is detected in the pit where endocytosis occurs, as observed using confocal microscopy, enabling the study of endocytosis in real-time. Also, dynamin 2 is a protein that mediates the endocytosis of vesicles from clathrin-coated pits. These molecular processes have been mapped using hopping SICM by mutating the gene coding for dynamin 2 and imaging the effect of this mutation on the morphology of the cell membrane undergoing dynamin-mediated endocytosis.^82^

**Figure 7:**
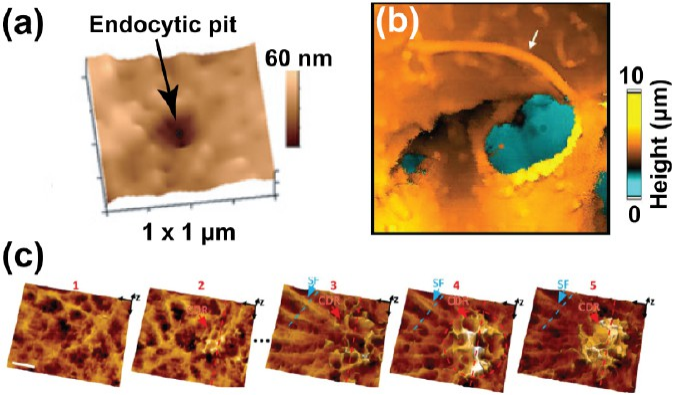
Using SICM for cell imaging of the membrane, and membrane structures relative to the cell membrane. (a) SICM image of clathrin-coated pit imaged on cell surface. Figure was adapted from ref^82^. Available under a CC-BY 4.0 license. Copyright 2019 Ali et al. Published by John Wiley and Sons. (b) Mapping of cilia on a Madin-Darby canine kidney (MDCK) cell, as shown by the white arrow. Figure 7b was adapted with permission from ref^85^. Copyright 2018, American Chemical Society. (c) The imaging of CDR on the surface of kidney fibroblast-like cells (i.e., COS-7 cells) using SICM. Figure was adapted with permission from ref^44^. Available under a CC-BY-NC-ND 4.0 license. Copyright 2021 Leitao et al. Published by the American Chemical Society. https://pubs.acs.org/doi/10.1021/acsnano.1c05202.

Interestingly, cilia have been studied by combining hopping-SICM (a noninvasive technique) and fluorescence microscopy, which reveals specific cellular activities on the cell membrane.^85^ The membrane-bound structure, the cilium, is a cytoskeleton composed of microtubules that play a structural role in the cell membrane. Using SICM, the pocket of the cilium has been examined (Figure 7b), which is impracticable using only fluorescence imaging. Recently, the dynamic monitoring of circular dorsal ruffles (CDRs), which are actin-rich structures formed on stimulated cells with growth factors, was captured on the surfaces of cell membranes using SICM (Figure 7c).^44^ CDRs are implicated in cellular activities, such as cell migration, receptor internalization, cancer propagation, and pathogen infection.^86–88^ These crucial roles of CDRs made them receive attention in the scientific community.^89^ SICM has revealed that without any stimulation of cells, CDRs can be captured using time-lapse studies, unlike other studies that revealed the detection of CDRs upon stimulation with mitogenic factors (Figure 7c).^44^

The inherent ability of SICM to measure the topography of living cells has been utilized in the study of live cardiovascular cells, which will enable the understanding of cardiovascular disease mechanisms at the tissue, cellular, and subcellular level. For example, it was revealed using SICM that loss of t-tubules in cardiomyocytes is associated with heart failure.^31^ Cardiomyocytes are known to have compartmentalized ion channels such as sodium and calcium channels. Existing patch clamp and immunofluorescence techniques have been used for the study of these types of channels on the cell membrane. However, the inability of these techniques to detect functional ion channels has posed challenges in establishing their functions. A study by Bhargava et al., described the development of a super-resolution scanning patch clamp.^90^ The technique coupled SICM with a cell-attached patch clamp, where a nanopipette is first used in SICM to measure the topography of cardiomyocyte, after which the nanopipette tip is moved to a new surface on the Petri dish without cells, where the tip is clipped against the Petri dish surface, resulting in an increase in the diameter of the tip. The clipped tip is then taken back to the surface of the original cell, followed by attaching it to the cell membrane to allow the measurement of ion channels. SICM-patch clamp has allowed the study of the activity of L-type calcium channels on the T-tubule of cardiac myocytes (Figure 8a–f, concept of SICM-patch clamp).^91^ This shows that SICM can map topography with high spatial resolution to guide in the identification of regions on cells containing ion channels.

**Figure 8:**
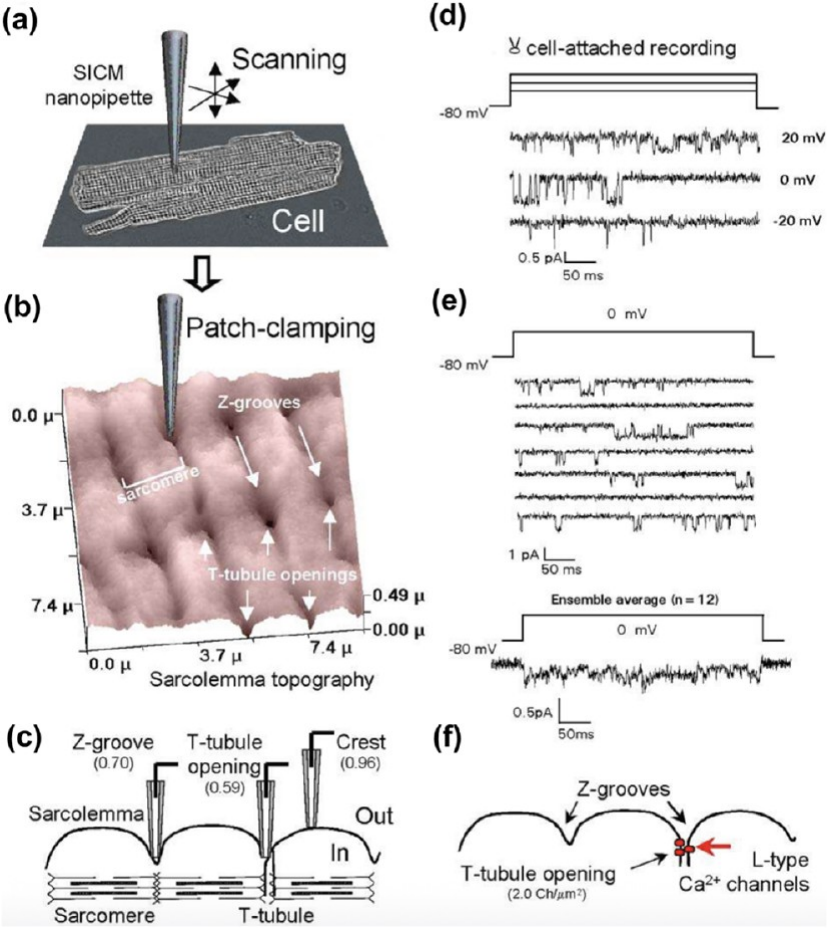
SICM-patch clamp for studying the topography and ion channels of cardiac myocyte, where (a) is the cardiac myocyte sarcolemma, (b) is the SICM topography of the cells showing that after SICM imaging, the nanopipette is placed on the T-tubule to measure the activity of L-type Ca^2+^ channels, (c) shows different SICM topographical regions where patch clamp measurements were performed, (d) shows the current traces as a result of L-type Ca^2+^ presence in the T-tubule, (e) multiple single-channel Ca^2+^ current recordings obtained from an individual patch, along with the corresponding ensemble-averaged current, and (f) shows the statistical distribution of detected L-type Ca^2+^ channel activity. Figure 8 was reproduced with permission from ref^91^. Copyright 2002, FASEB.

### 3.2 Interactions of Viral Particles with the Cell Membrane

Viral-like particles (VLPs) are nanoscale structures made up of assembled viral proteins without the viral genetic material, making them noninfectious.^92^ These particles are produced in numerous biological systems, including mammals, plants, insects, and bacteria.^93^ VLPs can be exploited as carriers for the delivery of bio and nanomaterials, such as drugs and vaccines.^94,95^ VLPs are gaining popularity in the field of preventive medicine, and to date, a wide range of VLP-based candidate vaccines have been developed for immunization against various infectious agents, such as the recent vaccine against SARS-CoV-2.^96^ Furthermore, VLPs are highly immunogenic and can elicit both antibody and cell-mediated immune responses by pathways different from those elicited by conventional inactivated viral vaccines.^97^

Techniques such as fluorescence confocal microscopy, scanning electron microscopy, and transmission electron microscopy are commonly used in the studies of viruses. However, limited information is obtained about the passage of nanoparticles through the cell membrane using fluorescence microscopy alone. The use of electron microscopy requires the use of an electron beam and the fixation of cells, which could potentially affect studying live cells and obtaining functional information.^98^ A study by Shevchuk et al., revealed that using SICM and confocal fluorescence imaging, termed scanning surface confocal florescence microscopy (SSCM), a distribution of VLPs on the surface of COS-7 cells were observed (Figure 9a–d), which suggests possible interaction with the cell membrane, but the uptake of the VLPs were not observed.^99^ The introduction of fluorescence microscopy into the SICM setup is to ensure that the native structures on the surface of cells can be discriminated from VLPs.

**Figure 9:**
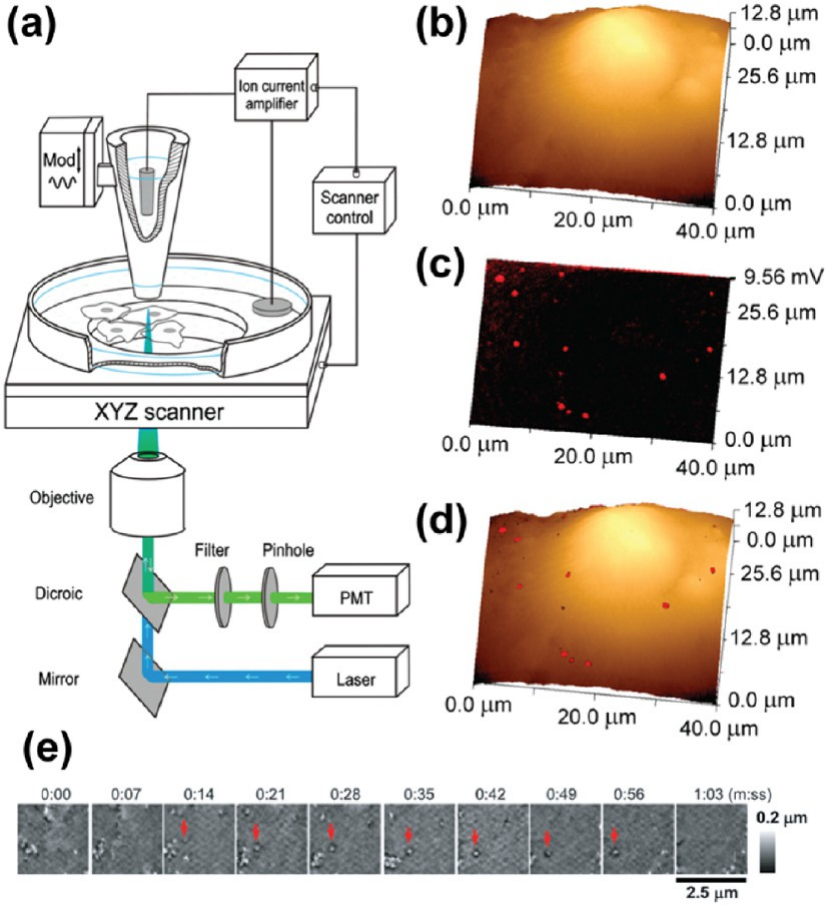
SICM for imaging nanoparticle interactions with the cell membrane. (a) Schematic showing the SSCM setup. (b) SICM image of the membrane of a COS-7 cell. (c) Fluorescence image of polyoma viral-like nanoparticles. (d) Using SSCM to image the interaction COS-7 with polyoma viral-like particles. Figures a–d were adapted with permission from ref^99^. Copyright 2008 Elsevier. (e) Timelapse imaging of HIV-like particles on the surface of Jurkat cell membranes using SICM-fluorescence confocal microscopy (SICM-FCM). Red arrows show the assembly and disassembly of the HIV-1 like protein in the same location on a cell membrane. Figure was reproduced from ref^100^. Available under a CC-BY 4.0 license. Copyright 2020 Bednarska et al.

The mean width of VLPs on a glass surface were characterized to be 115 ± 14 nm, while the ones on the membrane of COS-7 cells were 108 ± 16 nm, and when combined with fluorescence microscopy, it was revealed that the particles were VLP.^99^ Beyond studying VLP interactions with the cell membrane, the technique could be used for studying the interaction of a fluorescently labeled gene delivery agent with the cell membrane, which is crucial in the development of gene therapy agents. Recently, Bednarska et al., reported the use of SICM-fluorescence confocal microscopy (SICM-FCM) to understand the assembly of HIV-like particles, by recording the cell topography changes during the particle assembly on top of the cell membrane.^100^ This is in contrast to the traditional method that uses total internal reflection fluorescence microscopy (TIRFM), which relies on using the bottom surface of the cell in contact with the glass coverslip, instead of the top of the cell membrane. Using SICM-FCM, it was shown that the HIV-like particles were assembled into their full size within seconds, and released within 0.5 to 3 min on the cell surface of COS-7 cells, Jurkat cells (Figure 9e), and HEK-2932T cells, compared to an ∼ 8 min assembly time and 30 to 60 min release times that were previously reported using TIRFM. This method helped to challenge the current understanding of HIV replication, and can be used to study models for HIV replications.

### 3.3 Imaging Amyloids on the Surface of Cell Membranes

Biomolecules are constituents of living systems, which include carbohydrates, proteins, lipids, nucleic acids, and their respective monomers. For example, proteins are complex biomolecules that play key roles in cell membrane processes such as signaling and transport.^101^ Amyloid is a protein biomolecule that has been implicated in diseases such as type II diabetes, Alzheimer’s, and Parkinson’s.^102^ The mechanism by which amyloid proteins trigger these diseases is not yet fully understood.^103^

Development of effective therapeutics to target these diseases requires understanding of the assembly of amyloid fibril proteins. The application of AFM in the study of the morphology of amyloid fibrils is well established.^104^ However, the drawback of using AFM is that due to the contact between the cantilever tip and the cell membrane, there is damage to the amyloid fibril proteins. SICM has the potential to reveal the nanoscale structure of amyloid fibrils under physiological conditions.^105^ Studies by Zhang et al. revealed the structure of glucagon fibrils on a solid substrate using AC-SICM.^106^ The noninvasive properties of the AC-SICM were investigated with polydimethylsiloxane (PDMS), after which the AC-SICM was used to elucidate the morphology of glucagon fibrils on a surface. AC-SICM showed the self-assembly of glucagon into a mutant fibril, with spatial resolution obtained by SICM superior to that of ambient tapping mode (TM)-AFM.

Recently, SICM was used to image β-amyloid 1–42 (Aβ42) on the cell membrane of different cell lines (Figure 10).^107^ Correlative imaging using SICM and fluorescence microscopy revealed colocalization of Aβ42 structures, and fluorescently labeled fluorescein amidite (FAM)-Aβ42 further confirmed that the formed structures are amyloid aggregates. The interaction of the amyloids with the cell membrane induced cytoskeletal modifications, such as microtubule disassembly and actin polymerization, leading to a decrease in membrane potential and reactive oxygen species (ROS) generation. A limitation of studies of this type is that a direct causal mechanism linking β-amyloid aggregate formation on living cell membranes to specific cellular dysfunctions has not been established, beyond the observed correlations.

**Figure 10:**
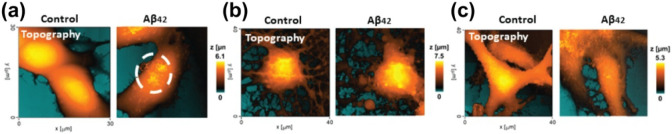
SICM mapping of amyloid proteins on the surface of the cell membranes of (a) SH-SY5Y cells, (b) hippocampal neurons, and (c) astrocyte cells. Figure was adapted with permission from ref^107^. Copyright 2023 American Chemical Society.

The interaction of different concentration of α-Synuclein (α-Syn) preformed fibrils (PFF) with neuroblastoma cells has resulted in increase in membrane roughness and presence of crystalline protrusion.^108^ The findings of the study highlighted the buildup of PFF on the cell membrane over the 48-h treatment period, which is characterized by increasing in roughness, while not triggering significant cell death. This study is significant in elucidating the mechanism of Parkinson’s disease. However, the interaction of PFF with normal cells has not been shown.

## 4 Mapping Mechanical Properties of Cells

### 4.1 Studies of Young’s Modulus of Cells Using SICM

Mechanical properties studies are an important aspect of mechanobiology,^109^ where studying the mechanical properties of normal and cancer cells can help unravel the pathology of diseases.^110^ The cytoskeleton is an important protein network that plays a crucial role in mechanical structure and cell motility, such as maintaining balance (as seen in blood–brain barrier), cell division (polymerization and depolymerization of microtubules), differentiation, and cell–cell adhesion (such as in tight and gap junctions).^111^ Changes in the cytoskeleton mechanical structure might be associated with disease conditions.^112^ The mechanical structure of cancer cells in promoting cancer has been extensively studied.^113^

Techniques such as AFM and other rheological microscopies are widely utilized in the study of the mechanical structure of cells, largely determined by the cytoskeleton.^114,115^ Recently, SICM has been developed for studying the mechanical properties of cells by Kolmogorov et al., where they revealed the use of SICM for topography and quantitative nanomechanical (QNM) mapping of PC3 cells (Figure 11a).^116^ The quantitative nanomechanical properties of cells were determined by measuring cell topography at different percentages of ionic current. Depending on the deformation (from intrinsic force) of the membrane at each set point, the Young’s modulus (stiffness) of cells was measured. SICM shows that treatment of PC3 cells with drugs (cytochalasin D and monomethyl auristatin) resulted in a decrease in the Young’s modulus, which correlates with a decrease in the stiffness of the cell, due to the inhibition of cytoskeleton polymerization.^116^ Apart from the use of hydrostatic pressure in nanopipettes to study stiffness, Rheinlaender and Schaffer have also reported the use of electroosmotic flow (EOS) in nanopipettes for mechanical peripeties studies.^117^

**Figure 11:**
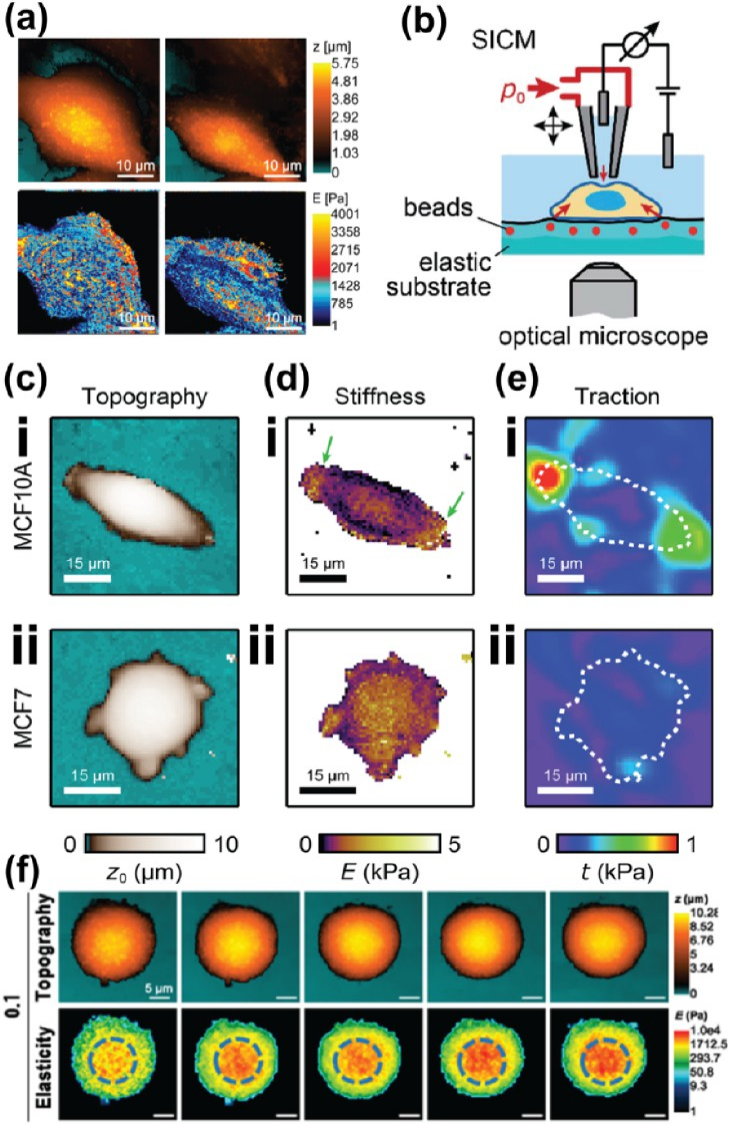
Use of SICM for mechanobiology studies of cells. (a) Using SICM for topography (top) and quantitative nanomechanical (bottom) mapping of single cells. Figure a was adapted with permission from ref^116^. Copyright 2021 Royal Society of Chemistry. (b) Schematic showing the setup for topography, mechanical properties, and traction force mapping in single cells. (c) Topography, (d) stiffness, and (e) traction force maps of (i) normal (MCF10A) and (ii) cancer (MCF7) cells. Figures b, e were adapted with permission from ref^119^. Available under a CC-BY 3.0 license. Copyright 2021 Rheinlaender et al. Published by the Royal Society of Chemistry. (f) Mechanical properties of CRC cells treated with H_2_O_2_. All scale bars in f are 5 μm. Figure f was adapted with permission from ref^120^. Copyright 2021 Elsevier.

More recently, Wang et al. developed an optically correlated pressurized SICM setup for mapping the mechanical properties of red blood cells (RBCs) exposed to diamide.^118^ The optical correlation allowed the sequential targeting of cells before and after inducing the RBCs. Using an ion current of 0.5% less than the bulk, the topography was mapped first, followed by 2% set point for stiffness, revealing the stiffness of RBCs increased by 4.9-fold after treatment with diamide, which is also characterized by decrease in indentation. The ion current signal in SICM-mechanical mapping is extremely sensitive to tiny changes in pipet-sample distance, making it difficult to separate true mechanical deformation from artifacts caused by pipet positioning, drift, or surface curvature, therefore, precise control and accurate correlation are essential.

It is unclear whether studying the stiffness of cells will give information about a disease.^121,122^ Therefore, there is need of correlating stiffness with parameters that signify cell behavior. Rheinlaender et al., developed an SICM technique (Figure 11b–e) that combines SICM (for topography and stiffness measurement) with traction force microscopy (for the studies of traction forces of cells).^119^ This technique was used to simultaneously measure the topography, stiffness, and traction force of normal Michigan Cancer Foundation-7A (MCF-7A) cells and cancerous MCF-7 cells. For normal cells as the stiffness increased the traction force increased, while for cancer cells there was no correlation between stiffness and traction (Figure 11c–e). This approach demonstrated the use of a single platform for studying multiple mechanical properties of single cells. Early studies by Kruztke et al. revealed the correlation between volume changes and stiffness,^123^ demonstrating the use of these two parameters to study cell properties.

Recently, Wang et al. have investigated H_2_O_2_ eustress (beneficial stress) in colorectal cancer (CRC), using SICM for mechanical properties studies.^120^ H_2_O_2_ eustress in cancer cells is critical for cancer progression. Most clinical trial H_2_O_2_ regulators in cancer cells not only failed to decrease H_2_O_2_, but mediated the progression of cancer.^124^ Exposure of CRC cells to H_2_O_2_ resulted in an increase in intracellular and extracellular H_2_O_2_, with an increase in cell stiffness, as shown using SICM (Figure 11f).^120^ The understanding that H_2_O_2_ eustress in CRC cells depends on the adaptability to increase in stiffness could enable the development of therapeutics to interfere with redox processes in CRC and other diseases that rely on H_2_O_2_ eustress.

### 4.2 SICM Methods for Correlating Cell Stiffness and Fluidity

Membrane fluidity is a biophysical property of the cell membrane, which plays a crucial role in cellular processes such as in endocytosis, cell development, and membrane fusion.^125^ Rheinlaender and Schäffer have developed an SICM method for the measurement of cell topography and fluidity, where they found that stiffness of a cell is inversely proportional to its fluidity, meaning the higher the stiffness the lower the fluidity and vice versa (Figure 12a–c).^126^

**Figure 12:**
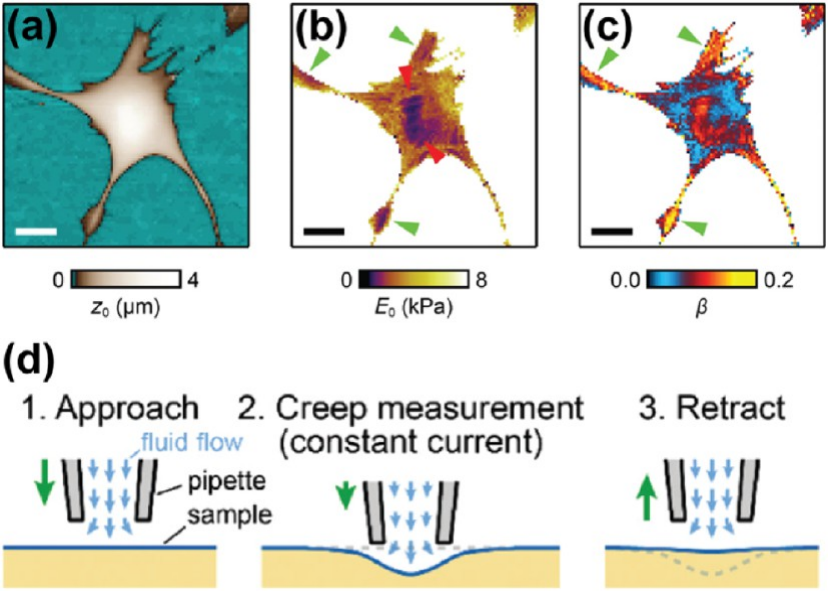
Fluidity mapping in single cells using SICM. Mapping of (a) topography, (b) mechanical properties, and (c) fluidity of single cells using hopping SICM. (d) Schematic showing the SICM method for measuring the fluidity, in which first the height of the cell was measured, followed applying a pressure of 10 kPa to the membrane for mechanical properties measurement (*E*_0_), and finally the nanopipette is retracted. The data from the height and mechanical properties measurement were then used to calculate the fluidity, β. Scale bars: 15 μm. Figure was adapted with permission from ref^126^. Copyright 2021 American Chemical Society.

To measure the topography and fluidity of a single cell, a nanopipette with an applied pressure of 10 kPa, is approached from the bulk solution to the cell surface, until there is 2% decrease in current, where the distance dependent force induced by fluid flow increases. Using another feedback loop, the ion current is set to deform the cell surface at 5% decrease in current, which enables measurement of constant force at the cell surface (Figure 12d). At the cell surface, the *z* position of the nanopipette decreases due to the viscoelastic property of the single cell, and then the nanopipette is retracted after measuring the viscoelastic property. To measure the fluidity, the cell deformation, δ­(*t*) is first calculated as shown in eq 12. The deformation is related to several parameters such as the distance of the nanopipette when the current is 1% less than the bulk current, *z*_0_, the distance of the nanopipette during the creep measurement at a current that is 2% less than the bulk current, *z*(*t*), and a constant, δ_0_, which depends on the pipet geometry, including parameters such as the tanα and the *r*_i_. The deformation is further related to the creep compliance of a cell, *J*(*t*), as shown in eq 13, where α is another constant that also depends on nanopipette geometry, and *p*_0_ is the constant pressure of nanopipette. The fluidity of the sample can then be determined using eq 14, where *J*_0_ is the cell compliance at *t* = *t*_0_, where *t*_0_ = 1 s, and β is the fluidity of the cell.

12 $$\delta \left(t\right)=z_{0}-z\left(t\right)-\delta_{0}$$

13 $$\delta \left(t\right)=\alpha p_{0}J\left(t\right)$$

14 $$J\left(t\right)=J_{0}{\left(t/t_{0}\right)}^{\beta }$$

SICM enables noncontact nanoscale mapping of cell mechanics but remains limited by slow imaging speeds, model-dependent Young’s modulus estimation, and calibration uncertainties.Also, SICM throughput, spatial resolution, and quantitative accuracy are lower than force-based methods like AFM, restricting broad or rapid mechanical analysis.

## 5 Functionalized Nanopipettes for High Spatial Resolution Sensing at Single Cells

### 5.1 Single-Barrel Nanopipettes Modified with Aptamers and Antibodies for Biosensing, and Future Directions for Their Applications in Single-Cell Analyte Imaging with High Precision

Functionalized nanopipettes are a powerful tool that can be used for a variety of applications, such as the detection of biomolecules and single-cell studies.^6,127^ Nanopipette tips are typically functionalized through a multistep surface immobilization process, with antibodies or aptamers that are selected to bind to target biomolecules.^32,128^ Nanopipettes functionalized with aptamers have been used for neurotransmitter detection. For example, a serotonin-aptamer-modified nanopipette sensor has demonstrated specific and selective responses to serotonin for real-time sensing applications.^129^ To ensure that each step of the fabrication processes of the aptamer functionalized nanopipette is completed successfully, a rectification coefficient (eq 15) is used as an indicator of nanopipette responses to changes in concentration, which are due to the fixed charges on the sensor surface.^130^ Monitoring the change in ion current rectification (ICR) after modifying the nanopipette allows for necessary tuning to achieve an optimal sensor, and gives validation of a successfully functionalized nanopipette. Apart from the advantage of easy and cost-efficient fabrication, nanopipettes functionalized with biorecognition elements have been proven to reversibly bind to their target analytes, allowing for real-time measurement, and multiple uses of the same nanopipette for detection of target molecules.^130,131^

15 $$r=\log_{10}\left|\frac{I_{+}}{I_{-}}\right|$$

To date, there are no reported studies that showed the use of aptamer or antibody functionalized nanopipettes for high spatial resolution imaging of an analyte concentration. Using these functionalized nanopipettes in SICM could allow for the simultaneous imaging of topography and an analyte concentration in real time in live cells. The advantage of using an emerging nondestructive technique (i.e., SICM) to take measurements, besides the ability to map analyte concentration and topography simultaneously, is that analyte delivery could also be facilitated without compromising the structural integrity of a sample.

### 5.2 SICM for the Simultaneous Imaging of [H^+^] Concentration and Topography in Single Cancer Cells Using Proton-Sensitive Nanopipettes

Multifunctional nanopipettes were developed for the simultaneous measurement of topography and [H^+^] concentration imaging, with high spatial resolution, however, they were not initially applied for pH sensing in single cells.^132^ For example, Nadappuram et al. developed a double barrel nanopipette, where one barrel was carbon pyrolyzed and IrO_2_ functionalized, while the other barrel was filled with KCl solution for topography mapping.^132^ This probe was used to reveal the pH distribution and the topography of calcite microcrystals, where the pH measured at the sample was more basic relative to the bulk solution. Morris et al. developed a nanopipette in which the outer surface was modified with gold to form a microelectrode, while the inner orifice opening was used for measuring membrane topography.^133^ This microscale pH-SICM probe was used to approach toward a porous polyimide membrane and then measure its local pH, where pH changes were detected on the surface of the membrane.

Measurement of cellular pH with high spatial resolution, accuracy, and precision will guide in the understanding of the implication of pH in diseases.^134^ Other methods of measuring the pH of single cells and tissues with high spatiotemporal resolution exist. For example, Huo et al. developed bright-field microscope-based UV–vis microspectroscopy that used common pH indicators (bromocresol green and bromothymol blue) to measure intracellular pH (pH_i_) in cancer and normal cells.^135^ The bromocresol green gives rise to a yellow monoanionic form in acidic media and a blue dianionic form in basic media, while bromothymol blue turns bright yellow under acidic conditions and dark blue under basic conditions. The treatment of cancer cells with either of these indicators, together with optical microscopic imaging, revealed cells exhibiting a yellow color, which indicates the acidity of the extracellular environment of cancer cells, while noncancer cells showed a dark blue color. Although pH can be mapped using dyes, this might be toxic to cells and thus limits measurements using live cells.^136^

Using endogenous and exogenous magnetic resonance active compounds, the pH of tissues has been measured. However, magnetic resonance imaging (MRI) and magnetic resonance spectroscopy have a low spatiotemporal resolution, and cannot determine the pH at the cellular level.^137^ Anderson et al., developed another technique for the measurement of acidic pH outside cancer tissues.^138^ The technique uses a pH-sensitive dye called seminaphtharhodafluor (SNARF). This dye is conjugated to the N-terminus of an insertion peptide which helps in the delivery of the SNARF dye across the cell membrane, depending on the membrane pH. The N-terminus of the peptide gets inserted into the cell membrane when its aspartic and glutamic acid residues are protonated. This pH dye conjugated to a peptide is called pH (low) insertion peptide (pHLIP)-SNARF, and was used to measure extracellular pH (pH_e_) of metastatic and nonmetastatic tissues.^138^ However, the use of pHLIP-SNARF for the measurement of pH at single cells is not possible, while the ease of proton diffusion in and out of cells makes it challenging to map pH in the cellular microenvironment by label assisted techniques.

Recently, Zhang et al. highlighted that noninvasive techniques for high-resolution and label free pH measurement in single cells are required, and they also reported the development and application of zwitterionic nanopipettes in a SICM platform for the simultaneous topography and pH_e_ mapping in single cells with high spatial resolution (Figure 13).^139^ The zwitterionic nanopipettes were developed using a cross-linked glucose oxidase/poly l-lysine membrane within one of the channels of a double barrel nanopipette used for pH sensing, while the other barrel was filled with electrolyte solution for traditional SICM topography mapping (Figure 13b). The topography and pH_e_ of MCF-7 cancer cells was successfully mapped simultaneously using this type of pH-sensitive nanopipette, where it was revealed that the cell topography mapped has a more acidic pH than the Petri dish surface (Figure 13c).^139,140^ The pH_e_ seen in this study is in agreement with the known pH_e_ of cancer cells of around 6.4–7.0 (Figure 13a),^141^ while normal adult cells have pH_e_ of ∼7.4.^142–144^ The understanding of pH_i_ and pH_e_ can be used in the theranostics of cancer at an early stage.^135,145^ Using this type double barrel nanopipette, pH sensors could offer a platform for the simultaneous measurement of topography, pH_e_, and pH_i_ in live single cells, which is crucial for the understanding of diseases, as proton concentrations divergent from normal levels could potentially be a sign of cancer, stroke, and Alzheimer’s disease.^135,146,147^ SICM-based pH sensing enables nanoscale mapping of local proton gradients, but is limited by probe instability, calibration drift, ion interference, slow scan speeds and tip-induced perturbations.

**Figure 13:**
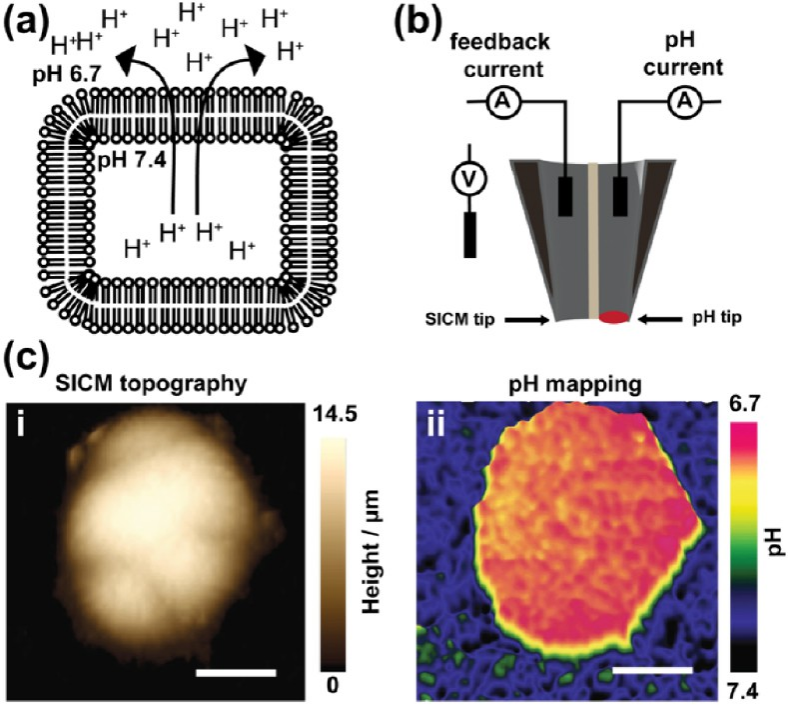
Extracellular mapping of proton concentration using SICM. (a) Schematic showing the pH_e_ and pH_i_ of cancer cells. (b) Nanopipette for use in SICM for the simultaneous mapping of pH and topography. (c) SICM topographic image (i) and pH map (ii) of MCF-7 cell. Scale bars: 20 μm. Figure c was adapted from ref^139^. Available under a CC-BY 4.0 license. Copyright 2019 Zhang et al.

## 6 Investigating the Effects of Biomolecule Perturbations on the Dynamic Changes in Cell Topography Using SICM

### 6.1 Dynamic Changes in Cell Topography upon Exposure of Cells to Bioactive Agents

Thrombin is known to trigger shape changes in platelets, as well as the generation of platelets activators such as serotonin, thromboxane A2, and growth factors.^148,149^ Seifert et al. reported the use of SICM for the study of morphology and dynamics of platelets activated by thrombin (Figure 14a,b).^150^ The advantage of SICM in platelets studies is that it avoids the mechanical stimulation of platelets, and it has been used to reveal dynamics of platelets after activation with thrombin, ensuring that the effects seen are due to thrombin, and not mechanical injury. This is unlike AFM, in which the tip makes contact with the cell membrane, which could potentially activate platelets, making it difficult to distinguish the effect of an activator (i.e., thrombin) and the instrument contribution in stimulation.^151^ Platelets that are activated with agonists other than thrombin did not show morphological dynamics, while activated platelets have shown dynamics of morphology (Figure 14).

**Figure 14:**
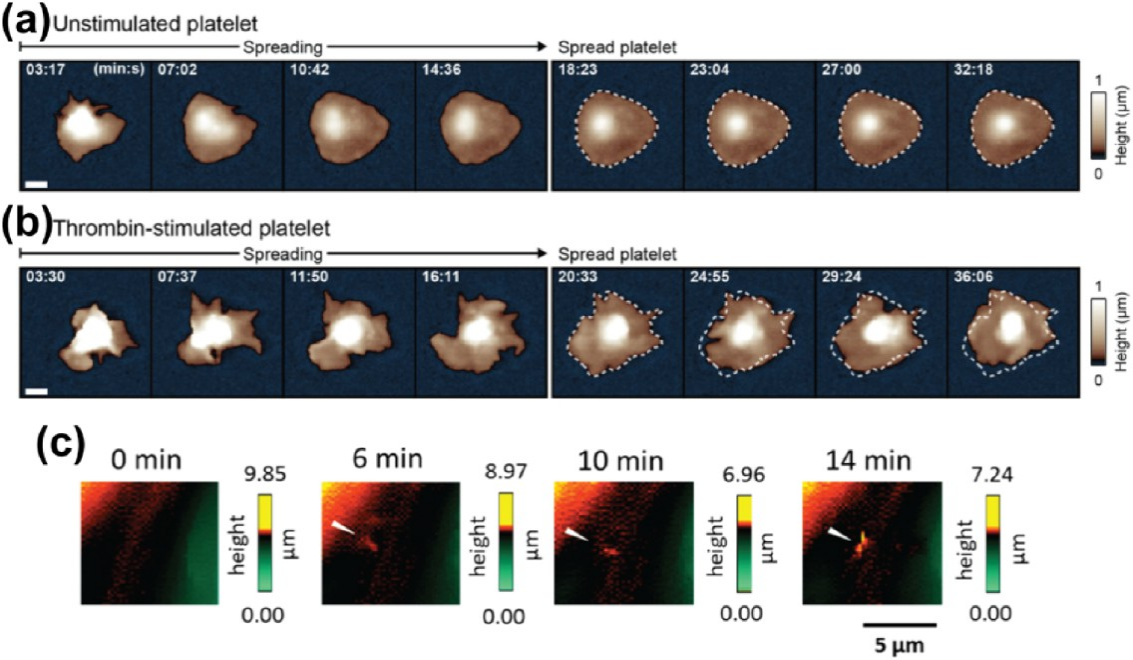
Changes in cell topography after exposure to thrombin and phorbol-12-myristate-13-acetate. (a) Morphology dynamics of unstimulated platelets not activated with thrombin during and after spreading. (b) The topography of platelets activated with thrombin during and after spreading. Scale bar for Figure a,b: 2 μm. Figures 14a,b were adapted from ref^150^. Available under a CC-BY 4.0 license. Copyright 2017 Seifert et al. (c) The studies of the exocytosis of vWF from human umbilical vein endothelial cells at different scanning times using SICM. Figure 14c was adapted with permission from ref^152^. Copyright 2015 American Chemical Society.

The interaction of cell membranes with a biomolecule that eventually leads to the release of proteins onto the membrane surface has been reported.^153^ Previously, AFM and fluorescence imaging have been applied for the studies of secretory proteins.^154^ However, immunofluorescent proteins potentially interact with the pore used for protein secretion and the molecule secreted from the pore,^155,156^ thus a technique that is noninvasive and label-free is required. Work by Nashimoto et al. using SICM highlighted that after growing human umbilical vein endothelial cell (containing Weibel-Palade bodies), and inducing it to secrete vWF using phorbol-12-myristate-13-acetate, a vWF protrusion was observed (Figure 14c).^152^ In the same study, opening and closing of the Weibel-Palade bodies for 18 to 30 min revealed that after 30 min, no protrusions were observed. Protrusions were also not observed in cells that were not stimulated to release vWF.^152,157,158^ The time scale of the exocytosis of vWF observed using SICM is comparable to that observed using fluorescence microscopy. The observation of strings and pore formation using SICM is innovative, as this is difficult using fluorescence microscopy methods.^152^ This revealed that SICM is a powerful tool for investigating the exocytosis in living cells.

### 6.2 Mapping Dense Core Vesicles Released by INSE-1 Cells Exposed to Glucose Perturbation

Insulin is a protein that is involved in the regulation of blood glucose.^159^ This protein is synthesized in the ribosome docked on the endoplasmic reticulum and transported to different Golgi networks for post-translational modifications, after which a regulated secretory vesicle is formed from the Golgi network containing insulin, which is then secreted outside the cell when glucose levels rise.^160^ Advances in fluorescence imaging such as super-resolution stimulated emission depletion (STED) and other biophysical methods have allowed the study of vesicle exocytosis. The use of fluorescence microscopy affects the kinetics of secretion, while biophysical methods provide less information.^161–163^ Using AFM to study exocytosis has been documented as challenging approach.^164^

A new method of studying insulin exocytosis was described by Bednarska et al., in which the topography of insulinoma cells (INS-1E), otherwise known as primary β-cells (capable of responding to 17 mM glucose and 0.2 mM tolbutamide by secreting insulin vesicles), was obtained using SICM and fluorescence microscopy (Figure 15).^165^ In this method, insulin vesicles were tagged with fluorophore to aid in SICM imaging at the precise location where exocytosis occurs. The SICM topography of the INSE1 cell showed the presence of membrane protrusions (microvilli and dorsal ruffles) after interaction with glucose, which confirms the role of glucose in the exocytosis of insulin granules as dense core vesicles (DCVs). The protrusion was proposed to be a vesicle containing insulin docked on the surface of INSE1 membrane.

**Figure 15:**
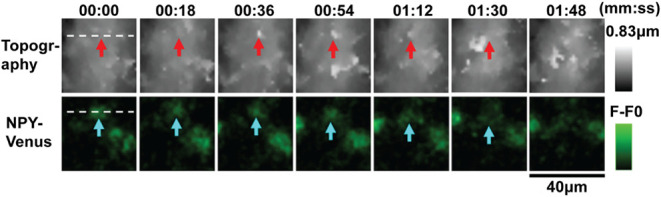
Timelapse dynamic change in membrane topography of INSE-1 cells displaying exocytosis of insulin-containing vesicles following exposure to glucose. Simultaneous fluorescence and SICM imaging to map glucose vesicle exocytosis. The top panels are the timelapse topographic images, before and after docking of the vesicle on the outer part of the membrane, while the bottom panels are the timelapse fluorescence images, showing intensity of the insulin vesicle when docking to the cytoplasmic and outer part of the membrane. Figure 15 was adapted with permission from ref^165^. Available under a CC-BY 4.0 license. Copyright 2021 Bednarska et al.

SICM has been successfully applied to visualize dynamic cellular processes such as thrombin-induced platelet activation, vWF release from endothelial cells, and insulin vesicle exocytosis in β-cells. These studies highlight SICM’s strength in providing label-free, high-resolution, live-cell imaging of nanoscale morphological changes during secretion events. However, the technique remains limited by low throughput, modest temporal resolution for fast exocytic processes, and the lack of direct molecular mechanism elucidation, requiring correlation with complementary techniques.

### 6.3 Dynamic Mapping of Cell Topography in Cancer Cells Treated with Anticancer Drugs and Antibiotics

Microscopic methods such as AFM, fluorescence microscopy, and SEM, are used for monitoring the morphology of cells induced with anticancer drugs. However, AFM could possibly damage cells, hindering it from use in dynamic studies in the same single cells over time.^44,166^ Fluorescence microscopy cannot be used for mapping cell topography and roughness, as the technique only relies on using fluorophores to tag molecules on the cell membrane.^167^ De Jonge and Peckys argue that the imaging of live cells using electron microscopy might be impossible.^168^ SICM is a highly appropriate technique for studying live cell morphology, and Muhammed and Lazenby used it in the study of apoptosis in live single lung cancer cells (A549), treated with the anticancer drug, cisplatin.^19^ The noninvasive nature of SICM allowed dynamic mapping of the same single cells’ volume, roughness, and topography over several days (Figure 16a,b).

**Figure 16:**
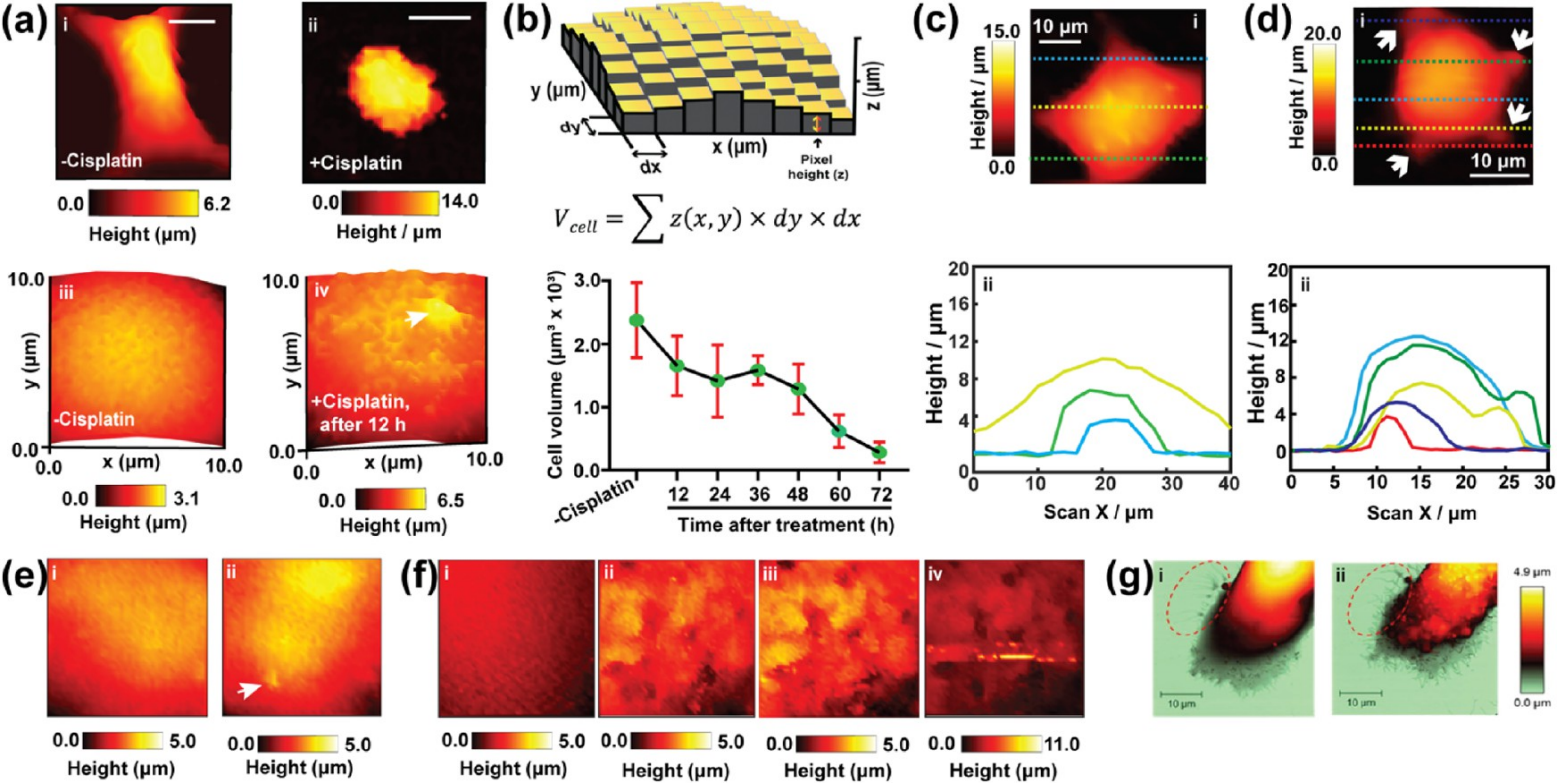
SICM for anticancer mediated apoptosis studies. (a) Morphological changes before (i) and after treatment with cisplatin (ii), and the changes in roughness of cell before (iii) and after cisplatin treatment (iv). (b) Apoptotic volume decrease studies in A549 cells treated with cisplatin over 3 days. Scale bar is 10 μm in part a. (c, d) The changes in whole cell morphology before and after toyocamycin treatment (i), and the corresponding line scans (ii). (e) The changes in membrane roughness of A549 cells (i) before and (ii) after treatment with toyocamycin, where no significant change in roughness was detected. (f) The changes in cell topography before (i) and (ii) 75 min, (iii) 80 min, and (iv) 92 min after treatment with toyocamycin, where the cells showed the formation of pits, associated with necrosis. Image dimensions for Figure 16e, f: 10 × 10 μm. Figures a, b were adapted from ref^19^. Available under a CC-BY 3.0 license. Copyright 2024 Muhammed et al. Published by the Royal Society of Chemistry. Figure c–f were adapted with permission from ref^169^. Copyright 2025 American Chemical Society. (g) Treatment of MCF-7 cells with plasma activated Ringer’s lactate solution. Figure was adapted with permission from ref^170^. Available under a CC-BY-NC 3.0 license. Copyright 2024 Usuda et al. Published by the Royal Society of Chemistry.

The shrinkage of morphology in A549 cells has also resulted in morphology rounding and blebbing (Figure 16aii), a characteristic feature of apoptosis. Also, treatment of the cells with cisplatin drastically changed the roughness of membrane of A549 cells. Using dynamic SICM, it was shown that the shrinkage in morphology of A549 cells is also correlated with a decrease in volume over time, where a significant decrease in volume after 60 and 72 h treatment with cisplatin was observed (Figure 16b). High resolution imaging of a small region of the cell membrane revealed the disappearance of the lamellipodia, and presence of membrane blebs around 2.6 ± 0.5 μm in diameter. The blebs measured were within the range reported using TEM.^171^ SICM is an invaluable tool for mapping apoptotic parameters such as cell body condensation, membrane blebbing, apoptotic volume decrease (AVD), and membrane roughness, with high temporal resolution. More recently, SICM has been applied for the mapping effect of toyocamycin on the morphology of A549 cells (Figure 16c,d), and studying the heterogeneous response of the cancer cells to the drug (Figure 16e,f).^169^ It was found that the toyocamycin induces apoptotic and necrotic behaviors in A549 cells by inducing membrane blebbing, cell shrinkage, both increases and decreases in volume, and pore formation on the surface of the cell membrane (Figure 16d–f).^169^ Using SICM, plasma-activated Ringer’s lactate solutions (PALs) were shown to activate apoptosis (Figure 16g), as indicated by an increase in height, a cease in cell migration, and formation of membrane blebbing.^170^

More recently, SICM has been used in the understanding of how antimicrobial peptides disrupt the membranes of cancer cells,^172^ using membrolytic-based disruptive mechanisms via electrostatic interactions (binding of an antimicrobial peptide to the negatively charged cell membrane). The binding of Temporin A with the surface of the membrane of A549 cells neutralizes the membrane’s negative charge, resulting in a steep approach curve when a nanopipette is approached onto the neutral surface. This binding creates a pit like structure on the membrane architecture, compared to a membrane surface that does not interact with Temporin A, as revealed by recording the topography using SICM. SICM excels in noncontact surface topography of live cells, but it provides limited direct information on biochemical, molecular or functional changes (e.g., signaling, ion flux, ROS) unless combined with other modalities. Recently, SICM has been used to study stiffness in drug resistant cancer cells, demonstrating reduced stiffness in resistant cells compared to normal ones.^173^ We believe that SICM could be an emerging tool for applications in drug discovery, especially when coupled with electrochemical methods, to allow simultaneous mapping of topography and sensing cell death biomarkers.

## 7 SICM for the Mapping of Topography, Cellular Delivery of Bioactive Compounds, and Biopsy

Researchers have used nanopipettes for the delivery of fluorescent biomolecules inside the cytoplasm.^174,175^ There is increasing interest in understanding the specific location of receptors on the cell membrane, such as heart cells.^176,177^ The localization of receptors on the surface of cell membranes may indicate their role in health, in which changes in the receptors may designate disease states.^158^ These changes can be studied using ion channel activation,^158^ or receptor stimulation.^90^ However, a technique that provides details on multiple phenomena is needed. Schobesberger et al. have used SICM to measure the topography of cardiomyocytes, in which the topographical features of the cardiomyocyte cell, such as crests and openings, were mapped with SICM using a −200 mV bias (Figure 17a).^178^ After the SICM imaging, the nanopipette probe was then used to apply 50 μM isoproterenol into opening of the cardiomyocyte, rich in membrane receptors, by holding the nanopipette at +400 mV (Figure 17a). The isoproterenol is a positively charged molecule, so the nanopipette positive voltage drives it outside the nanopipette. Also, isoproterenol is an agonist for the β_2_-adrenergic receptor, that results in receptor activation, resulting in a decrease in response as shown in Figure 17b, compared to Figure 17c,d where no response was detected when the isoproterenol was added to the crest region.

**Figure 17:**
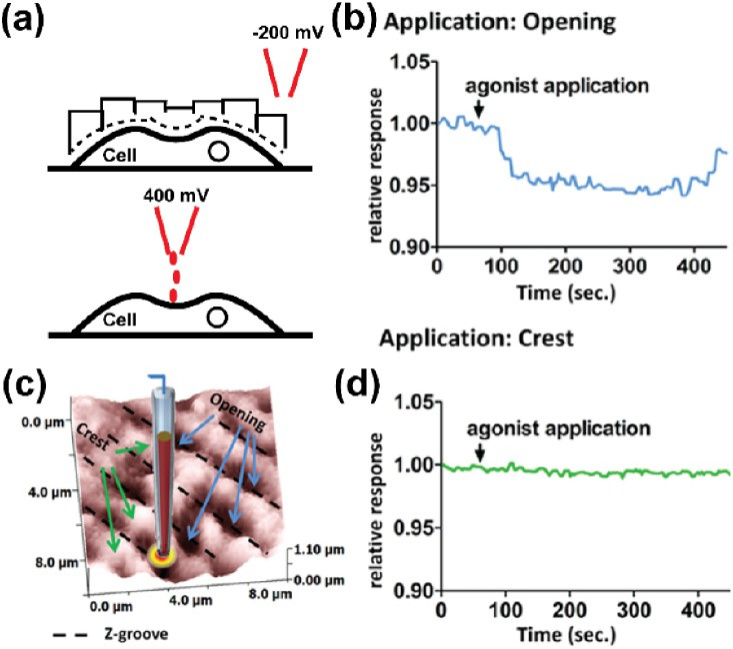
(a) SICM enables the revealing of the cardiomyocyte membrane structure, for the application of bioactive compounds. (b) Measurement of the topography of cardiomyocytes to detect crests and openings, followed by the application of isoproterenol into (c) the opening surface of the cardiomyocyte resulted in the activation of β_2_-adrenergic receptor, (d) while application of the compound to the crest does not result in receptor activation. Figure was adapted from ref^178^. Available under a CC-BY 4.0 license. Copyright 2016 Schobesberger et al. Published by Elsevier. https://doi.org/10.1016/j.bpj.2015.11.017.

This study revealed the importance of membrane topography measurement in the delivery of biomolecules to a single cell. This new method could open new opportunities in the study of intracellular biomolecules and pathways, without the need to rely on the endocytosis of dyes into the cytoplasm. This is especially the case when the process involves morphological changes, where SICM can be performed simultaneously before and after the intracellular delivery. SICM’s strength in combining high-resolution topography imaging with precise, localized delivery of molecules to individual cell sites is remarkable. However, it is limited by slow imaging speeds, low throughput, and challenges in achieving reproducible quantitative dosing due to electrokinetic and geometric variability.

Nanopipette positioning using SICM has enabled the ability to perform biopsies from single cells, allowing the analysis of intracellular material.^179–181^ This capability represents a significant advance toward genomic interrogation at the single-cell level. A key advantage of this approach is that the nanopipette can extract extremely small quantities of intracellular material. However, this also presents a major challenge, as the recovered volume may be insufficient for downstream analytical techniques, and requires amplification. In cases where amplification is not feasible, further analysis becomes challenging. Nonetheless, amplification methods such as polymerase chain reaction (PCR) can be applied to single-cell DNA to generate sufficient material for downstream studies.

## 8 Combining SICM with Scanning Electrochemical Microscopy (SECM)

An analytical tool with high spatial and temporal resolution, capable of operating under physiological conditions is essential for assessing the correlation between localized topography and biomolecular functions.^182^ Conventional SECM using microelectrodes is challenging in mapping surfaces that are not flat such as single cells.^183^ The major challenge associated with nanoscale SECM is incorporating some means of achieving constant distance imaging, to ensure the accurate measurement of analyte concentration close to a cell surface and to avoid the tip crashing into the substrate surface. The combination of SICM with SECM is a reliable way to allow topography and redox species mapping simultaneously on the cell membrane, as comprehensively discussed by Bard and Mirkin.^184,185^ Carbon nanoelectrodes provide nanoscale resolution for monitoring redox reactions, for example at the surface of thylakoid membranes.^186^ Also, carbon nanoelectrodes functionalized with Pt have been used for nonspatial resolve H_2_O_2_ measurement, as shown by Korchev and coworkers.^187^ Significant progress in live single cell imaging has been made in the field of carbon nanoelectrodes by Takahashi and coworkers, where they demonstrated the use of carbon pyrolyzed nanoelectrodes for spatial resolve detection of epidermal growth factor receptor (EGFR) on A431 cells, and neurotransmitters on hippocampus neurons.^183^

Takahashi et al. have also advanced carbon nanoelectrodes into ion conductance nanopipettes to detect topography and measure electrochemical flux in a combined SICM-SECM setup, where oxygen on the surface of cell membrane of cardiac myocytes was mapped (Figure 18a).^182^ Large oxygen reduction reaction currents were observed at the cell membrane, compared to the surface of a Petri dish (Figure 18b). This is an indication of high uptake of oxygen by single cells through diffusion.^182^ SECM is a powerful tool for estimating transport rates of electrochemically active species across interfaces, which makes it a useful technique to complement SICM.

**Figure 18:**
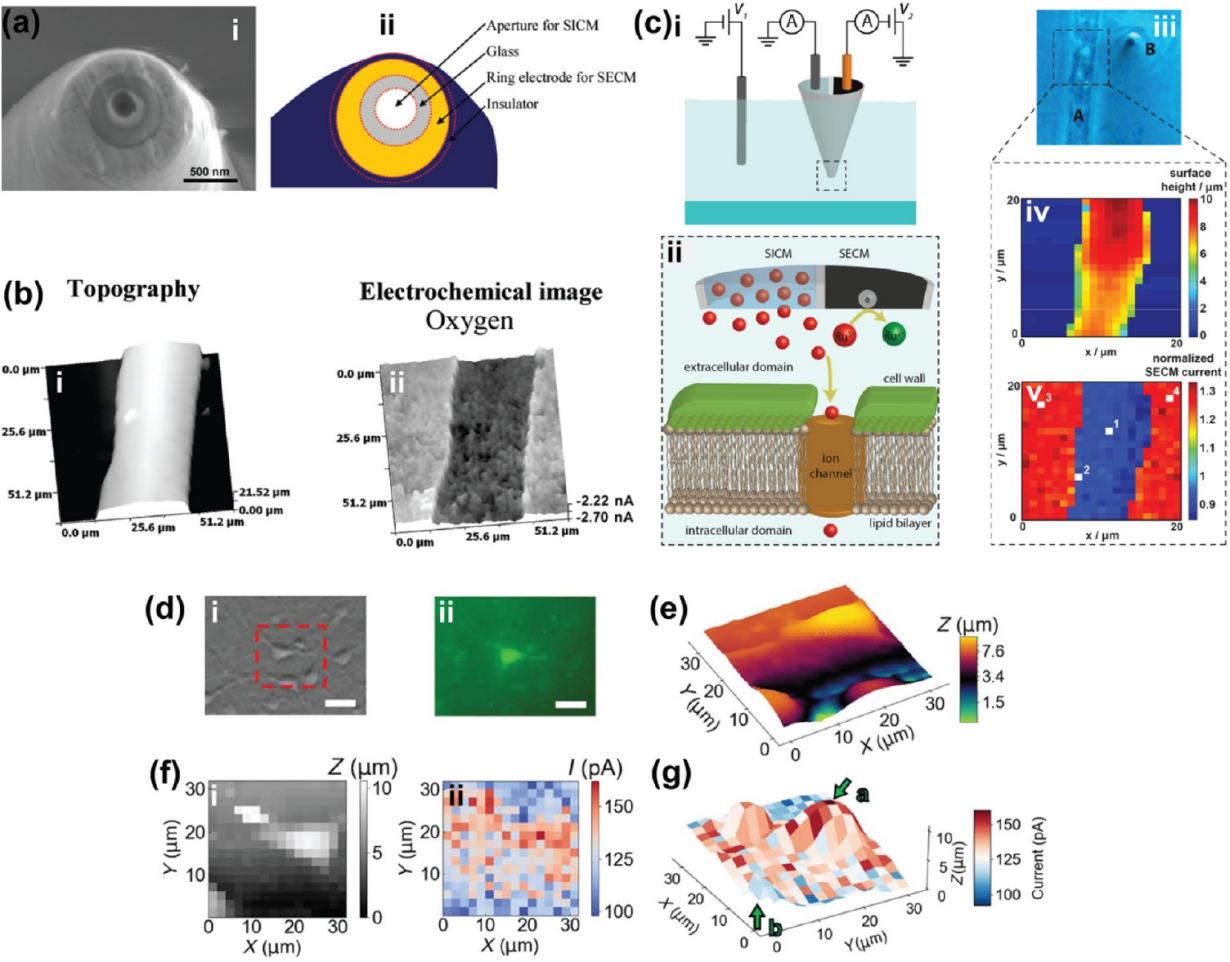
(a, b) Using SICM-SECM nanopipettes for the simultaneous mapping of topography and oxygen distribution on the surface of cardiac myocytes. Figure was adapted with permission from ref^182^. Copyright 2010 American Chemical Society. (c) (i, ii) SICM-SECM was used to deliver [Ru­(NH_3_)_6_]^3+^ to a maize cell (iii), and the topography (iv) and uptake of [Ru­(NH_3_)_6_]^3+^ (v) were mapped. Figure was adapted with permission from ref^188^. Copyright 2017 American Chemical Society. (d) Mapping a dopaminergic (DA) cell (left optical image and right fluorescent image), showing the (e, f) topography (left) and current (right) on the surface of neuronal cells using SICM-SECM, with an overlay of the redox current for DA oxidation on the topography show in (g). Figure d scale bar: 20 μm. Figure d–g were adapted with permission from ref^189^. Available under a CC-BY-NC 4.0 license. Copyright 2024 Wang et al.

Page et al., developed SICM-SECM probes for the simultaneous measurement of topography and uptake of analyte by *Zea mays* root hair cells adhered to a Petri dish. The SICM-SECM probe used was multichannel, where one of the channels was used for SICM feedback and filled with electroactive species (i.e., ruthenium hexamine) for use in generating current by the other channel that was a pyrolyzed carbon electrode (Figure 18c). During the SICM-SECM imaging, hexaammineruthenium­(III) ([Ru­(NH_3_)_6_]^3+^) flows from the SICM barrel to the electrode surface at the end of the carbon barrel, where it undergoes a reduction to [Ru­(NH_3_)_6_]^2+^, which generates a redox current. When the probe was positioned over the Petri dish, a high current was observed due to the hindered diffusion and increased concentration of [Ru­(NH_3_)_6_]^3+^ around the carbon barrel. Whereas, when the probe was over the root hair cell, a decrease in current was detected due to uptake of [Ru­(NH_3_)_6_]^3+^ by root hair ion channels (Figure 18ciii–v).^188^ This demonstrates the development of a noninvasive platform for the studies of ion uptake by single cells.

One of the most recent advancements in the use of carbon nanoelectrodes in SICM is fast scanning cyclic voltammetry (FSCV)-SECM-SICM, which was introduced by Baker and coworkers (Figure 18d–g).^189^ They reported the use of multifunctional nanopipettes, where one of the barrels was used for SICM (to map topography of the soma of cells), while the other barrel was used to generate a redox current when the neurotransmitter dopamine was released. They revealed that in a cocultured dish containing dopaminergic (DA) cells (GFP labeled) and non-DA cells (unlabeled) (Figure 18d), only K^+^ stimulated DA cells generate an oxidative current at +0.5 V. This corresponded to the oxidation of dopamine, as shown in the simultaneous acquisition of fluorescence, topography, and electrochemical images, while stimulating non-DA cells did not generate oxidative currents. Combining SICM with SECM enables simultaneous topographical and electrochemical mapping which introduces analyte mapping in SICM, but suffers from low spatial resolution, low probe fabrication success, and signal crosstalk between channels. More recently, SICM was performed sequentially with SECM to map the topography and H_2_O_2_ in single cells.^190^

## 9 Surface Charge Mapping using Nanopipettes in SICM

Understanding the local surface charge of cell membranes could reveal mechanisms of membrane binding, drug delivery, and the design of liposomes.^191^ Some of the common techniques that are used for surface charge mapping include Kelvin probe force microscopy and electrostatic force microscopy (EFM),^192,193^ although these have some limitations such as the need for nonporous, smooth and flat samples, and in the case of EFM the sharp probe can destroy a sensitive sample. SICM has allowed surface charge mapping, using sample with complex topography or high sensitivity, with high spatial resolution.^191,194^

An interesting phenomenon associated with using nanopipettes in SICM for mapping charged surfaces is ICR (vide supra), which arises when the size of the nanopipette tip orifice is a similar order of magnitude to the EDL.^195^ The effect is intensified for low salt concentrations, because the lower the concentration of the electrolyte the thicker the EDL will be. ICR results in a nonlinear current response observed in a voltammogram (*I*-*V* plot), which can be explained by the influence of the EDL on migrating ions through the nanopipette. For example, when a glass nanopipette is immersed in KCl solution with a pH of 6.8, the glass will possess a negative charge, which needs to be compensated by K^+^, which results in the formation of a EDL at the surface of the glass in which Cl^–^ is excluded from the layer formation. The EDL will interfere with ions migrating through the orifice, resulting in permselectivity of the nanopipette.^196,197^ A positive potential in the pipet will drive the migration of Cl^–^ inside the pipet, while K^+^ moves out of the pipet, whereas the ions will reverse direction under a negative potential.^195,198^ Under low electrolyte concentration, this affects the response of SICM when the nanopipette approaches a charged surface, and will also depend on the charge of the substrate due to its EDL. As the distance between the tip and the interface decreases, the current may increase or decrease, depending on the nature of charged interface and the polarity of the tip electrode.^199^ When the nanopipette is held at a negative potential and approached to a negatively charged surface, accumulation of negative ions will occur at the tip, due to the effect of the surface charge, resulting in an increase in negative current. Likewise, when the nanopipette is held at a positive potential and approached toward a positively charged surface, an increase in positive current will be observed. This concept has been used for surface charge determination.^17^

Recently, Cremin et al., mapped the surface charge of live bacterial cells, using two potential switching methods (Figure 19a).^200^ One of the modes is pulsed potential, where the nanopipette is approached to the substrate surface at 50 mV, and then the charge surface is mapped at −500 mV. This method was used to map the surface charge of Gram-negative bacteria (Figure 19c). Another method is approaching the nanopipette to a substrate at 50 mV, then cycling the voltage between −500 and 500 mV, which was also used to detect surface charge of both Gram-negative and Gram-positive bacteria (Figure 19b).

**Figure 19:**
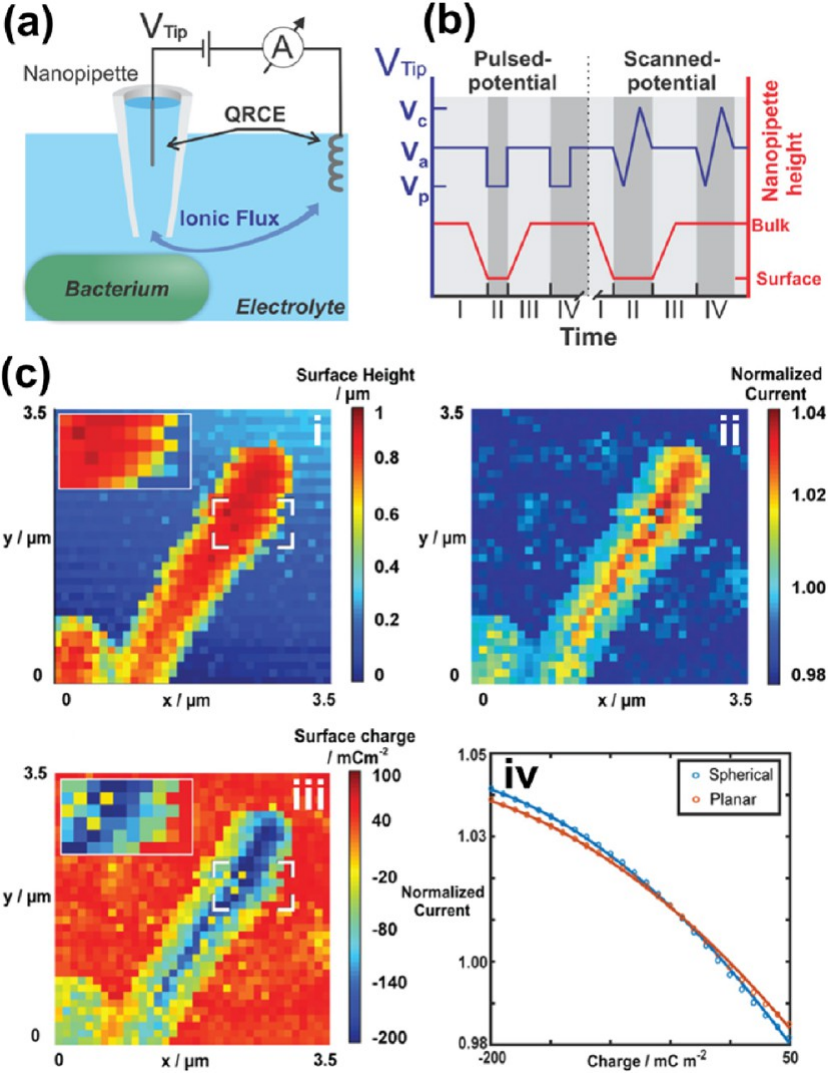
Surface charge mapping of live bacterial cells using SICM. (a) Schematic showing the use of nanopipettes for surface charge mapping in live bacteria cells. (b) Voltage switching methods applied to the nanopipette, to enable surface charge mapping. (c) Topography (i), normalized surface current (ii), surface charge of *E. coli*, which was generated from the SICM current by modeling *E. coli* using FEM as a planar charge insulating surface (iii), and the FEM simulation for the comparison of the charge variation in model used (planar vs spherical) for the conversion of current to surface charge, where there is only small difference in current between the two FEM models. Figure 19 was adapted from ref^200^. Available under a CC-BY 4.0 license. Copyright 2020 Cremin et al.

Other studies have also shown the use of hopping SICM for surface charge mapping in bacteria, *Pseudomona aeruginosa* PA14, where the charge density was reported to be −2.0 ± 0.45 mC/m.^2,58^ The imaging of charged cell surfaces might be challenging when using a single barrel SICM probe. As biological samples typically carry electrical charges in liquid environments, when the surface charge is present, the resulting ion current behavior during the approach curve is influenced by both the sample’s surface charge state and the distance of the nanopipette electrode from the sample. Using double barrel nanopipettes allows the desensitization of charged surfaces and makes it possible to map the topography and surface charge of highly charged cellular surfaces, such as chromosomes, which exhibit a negative charge in phosphate buffered saline (PBS) solution.^201^ This method that uses a double-barrel nanopipette enabled accurate measurement of chromosome surfaces, free from interference caused by surface charges. SICM-based surface charge mapping enables noncontact, high-resolution visualization of local electrostatic heterogeneity of hard and soft surfaces. However, it remains limited by model-dependent quantification, tip-geometry sensitivity, slow mapping speed, and environmental factors (ionic strength, pH) that complicate calibration and reproducibility.

## 10 Conclusions

There has been significant progress in SICM for cellular studies, such as the mapping of cell membrane architectures (for example insulin vesicles and membrane interactions with amyloids), mechanical properties (such as stiffness and fluidity), ion channels, delivery of molecules, biopsy, surface charge mapping, and mapping of membrane analyte concentrations, such as H^+^ (i.e., pH). Coupling SICM with SECM has enabled the simultaneous mapping of topography of cells and the measurement of neurotransmitters such as dopamine. Further advancement in the speed of SICM to map topography and electrochemical activity could enable measurement of fast biological events. SICM has also been reported for the studies of cancer cell interactions with antitumor drugs, and for high resolution mapping of the membranes of cells showing apoptotic and necrotic behaviors. Coupling SICM with electrochemical sensing could advance the technique for applications in drug discovery, where changes in topography can be correlated with changes in analyte concentration in single cells.

Advancements in SICM instrumentation have enabled the imaging of time-lapse dynamic cellular events, such as endocytosis, micropinocytosis, mitosis, bacterial infection, and cell differentiation in cancer cells. This could allow better understanding of biological events such as cell–cell interactions for infection, immunology, and cancer research, as high temporal resolution time-lapse measurements are crucial. The development of SICM-FCM to study HIV-like particle assemblies can be used to study models for HIV replication. The advancement in SICM in the delivery of biomolecules onto the cell membrane will offer opportunities in the delivery into the cell membrane without the need to rely on endocytosis. Among the emerging fields of SICM in single cell studies is introducing specific analyte detection into the nanopipette imaging probe, such as using pH sensitive materials like glucose oxidase/poly lysine or iridium oxide. Development of new SICM probes functionalized with other materials capable of detecting analytes of biological importance in single cells will be critical in the application of the technique in biological studies. SICM is a powerful noninvasive technique for high-resolution imaging of live cell surfaces, enabling detailed visualization of nanoscale morphology and dynamic processes without physical contact. However, it is limited by low throughput, modest temporal resolution, and the lack of molecular specificity. Coupling it with complementary techniques, such as fluorescence microscopy or electrochemical methods, can provide correlative structural-functional insights and overcome these limitations.
